# Unravelling taphono-myths. First large-scale study of histotaphonomic changes and diagenesis in bone from modern surface depositions

**DOI:** 10.1371/journal.pone.0308440

**Published:** 2024-09-26

**Authors:** Eline M. J. Schotsmans, Barbara H. Stuart, Tahlia J. Stewart, Paul S. Thomas, Justyna J. Miszkiewicz

**Affiliations:** 1 Environmental Futures Research Centre, School of Earth, Atmospheric and Life Sciences, University of Wollongong, Wollongong, Australia; 2 PACEA, De la Préhistoire à l’Actuel: Culture, Environnement et Anthropologie, UMR 5199, CNRS-Université de Bordeaux, Pessac, France; 3 Centre for Forensic Science, University of Technology Sydney, Ultimo, NSW, Australia; 4 Skeletal Biology and Forensic Anthropology Research Group, School of Archaeology and Anthropology, Australian National University, Canberra, Australian Capital Territory, Australia; 5 Trauma and Orthopaedic Research Unit, Department of Orthopaedic Surgery, The Canberra Hospital, Canberra, ACT, Australia; 6 School of Civil and Environmental Engineering, University of Technology Sydney, Ultimo NSW, Australia; 7 School of Social Sciences, University of Queensland, Brisbane, Australia; Seoul National University College of Medicine, REPUBLIC OF KOREA

## Abstract

The use of diagenetic alterations in bone microstructure (‘histotaphonomy’) as indicators of funerary treatment in the past and for post-mortem interval calculations in forensic cases has received increasing attention in the last decade. Studies have used histological changes to conclude *in-situ* decomposition, mummification, infanticide and post-mortem interval. There has been very little attempt to experimentally validate the links between decomposition, depositional conditions, time-since-death and microscopic changes in human bone so that meaningful interpretations of archaeological and forensic observations can be made. Here, we address this problem experimentally using the largest sample of human remains from anatomical donors and the longest-term deposition framework to date. This study tests one key assumption of histotaphonomy; that putrefaction during the early stages of decay is reflected in bone microanatomy and composition. Seventeen human donors and six pigs were deposited on the surface in a known Australian environment and left to decompose between 463 and 1238 days. All remains underwent all stages of decomposition reaching skeletonisation. Rib and femur samples were analysed using conventional histological methods and scanning electron microscopy, by applying the Oxford Histological Index, and examining collagen birefringence, microcracking and re- and de mineralisation. Biomolecular changes of the femoral samples were analysed using Fourier-transform infrared (FTIR) spectroscopy. The results indicate that bioerosion in human bone does not occur due to putrefaction. There were no correlations between bone histology and the following variables: human *vs* pigs, season, primary *vs* secondary deposition, position, fresh *vs* frozen and time-since-deposition. Furthermore, no trends were observed between biomolecular changes and time-since-deposition. The study also shows that pigs cannot be used as substitutes for human remains for bone biodegradation research. This is the first, controlled, larger scale study of human remains providing a lack of support for a long-assumed relationship between putrefaction and bone histology bioerosion. Using bone degradation as an argument to prove putrefaction, *in-situ* decomposition and early taphonomic processes cannot be supported based on the experimental human data presented.

## Introduction

Analysing human remains and their depositional context, can provide a wealth of information about the individual themselves, their lifestyle and mortuary practices of their society. In most cases, bone is the only tissue that survives time. In addition to macroscopic anatomical methods, microscopic analyses of bone histology have been used to study age-at-death, sex, behaviour, health, diet and disease both in ancient and recent human remains [1–4]. The study of diagenetic processes in archaeology, geochemistry and sedimentology gained momentum in the mid to late 20^th^ century, but the analysis and interpretation of diagenetic alterations in bone microstructure specifically, known as ‘histotaphonomy’, is a more recent development, increasingly studied from the 2000s onwards [5]. Bone diagenesis encompasses the physical, chemical and biological processes that alter or degrade bone [6, 7]. At a microscopic level, respective examples are cracking, de- and hyper-mineralisation and microscopical foci of destruction (MFD) created by micro-organisms [6, 8, 9]. Wedl [10] was the first to describe microscopic tunnelling in fossil bones and teeth, originally thought to be caused by fungi. The tunnels became known as Wedl tunnelling and are nowadays attributed to cyanobacteria, typical for aquatic environments [8, 11, 12]. Tunnels of bacterial origin were classified by Hackett [13] in budded, lamellar or linear longitudinal according to their microarchitecture and how they invaded the bone. While several authors [e.g. 14–16] continue to use these associate descriptive morphologies, limited research exists validating their origin in bone. Indeed, multiple alternative explanations as to their origin can be given, ranging from inherent taphonomic changes to external sources, such as technical preparation of histology sections, determining their appearance seen microscopically. Recently, Turner-Walker [8] concluded there is no convincing evidence that their different morphologies link with different bacterial aetiologies.

The most established method to score microscopic diagenetic alteration in bone is the Oxford Histological Index (OHI), originally proposed by Hedges et al. [17] and further developed by Millard [18], scoring the percentage of intact bone, unaffected by bioerosion. The OHI is often used in combination with bone birefringence scoring [13, 19]. More recently, a whole range of taphonomic scoring indices have been developed and re-developed [e.g. 15, 20–23] but their reproducibility has not been tested and several face challenges due to methodological subjectivity. For example, it has yet to be understood how researchers measure or judge ‘enlarged’ lacunae or canaliculi [14, 23–25] while they appear filled instead of empty, rather than enlarged.

All these unvalidated methods have one common goal; the reconstruction of post-mortem biographies of human remains. At the core of controversy in this matter is the origin of osteolytic microbiota. This has led to a dichotomy of opinions between endogenous and exogenous bacterial origins. On the one hand, it is thought that osteolytic bacteria originate from the depositional environment [16, 26–30]. In contrast, other researchers claim that bacteria originate from the gut [9, 15, 23, 31] and that ribs are more susceptible for biodegradation because of their location ‘closer’ to the intestines [9, 32–34]. Some researchers are more cautious and state that differences in bone diagenesis are difficult to explain. It is difficult to discern the many unknown factors related to the funerary conditions of the remains and the local microenvironment that change over time [8, 21, 35, 36].

The origin of the gut-soil discussion goes back to observations made by Jans et al. [9] that bones from articulated animal and human remains were more affected by bacterial degradation than disarticulated ones. Several authors have argued that bacterial degradation is linked to putrefaction and the early stages of decomposition based on arguments that butchered animals do not undergo the putrefaction stage [9, 23, 31], although this hypothesis does not take into account the treatment of butchered animals such as cooking or baking kills bacteria [8]. Observations of a lack of biodegradation in stillborn pigs [23] and a limited presence of bacterial attack in archaeological human infant bones [37] have been used as additional arguments to confirm the gut bacteria hypothesis assuming that ‘bioerosion reflects the extent to which remains are exposed to bodily putrefaction’ [23, 38].

Ever since, a myriad of researchers has gone further with interpretations by trying to deduce early post-mortem practices from histological thin sections, but without experimental validation of these histology-putrefaction assumptions, thus leading to interpretative inconsistencies reported in the literature. In 2015, Booth et al. [39] claimed that a lack of microscopic biodegradation could indicate mummification practices because “ancient mummified bones are unlikely to have been affected by putrefaction” (p1156). This statement implies a lack of understanding of mummification. Mummy studies have shown that mummies pass through the stage of heavy putrefaction before they desiccate [40, 41]. Further, Trueman and Martill [38] stated that early post-mortem processes such as the mode of death influence the potential of any bone to survive into deep time. Martinon-Torres et al. [42] reported presence of MFD in bone as an argument to identify the earliest known human burial in Africa, claiming that biodegradation in bone is evidence of putrefaction to support *in-situ* decomposition. Similarly, other researchers directly associate putrefaction with the presence of biodegradation in bone [e.g. 15, 43]. In contrast, some studies advise caution when using the absence/presence of bioerosion in human bone to assess ‘putrefaction’. Haddow et al. [14] could not distinguish individuals with clear archaeological and osteological indications of delayed burial on a histotaphonomic level and stressed that many other contextual variables might have played. Rather than putrefaction, Trenchat et al. [44, 45] link inter-individual differences in histological preservation to different biological parameters (primary bone) and different container use (zinc/wood coffins). Hemer et al. [46] observe different histological preservation caused by different post-mortem treatment of plague victims instead of putrefaction. And Eriksen et al. [11] and Turner-Walker [47] observe different aerobic and anaerobic bone bacterial communities between terrestrial and aquatic contexts. These last six references show the interpretative potentials of histotaphonomy and stress the importance of contextual information.

In forensic science, it has been claimed that bone bioerosion can be used as a method for post-mortem interval estimation, based on animal model experiments, such as those of a rat or a pig [22–24], and suggested that stillborn and neonatal death can be distinguished histotaphonomically [23]. However, these studies show low replicability and ignore biological variation in bone histology between humans and other mammals [48]. Flawed post-mortem interval (PMI) calculations and infanticide claims have serious implications for forensic investigations and thus should be conducted with methodological rigour [8, 48].

Another problem has been the single focus on analysis of histological sections with light microscopy [e.g. 15, 25, 49]. The use of only light microscopy is insufficient for the study of diagenetic changes in bone and low-resolution identifications might lead to misinterpretations of artifacts. To understand bone diagenesis holistically, microstructural and molecular changes should also be studied. Microcracking, de- and re-mineralisation, and the presence of fungi or sediment can be examined using electron microscopy [e.g. 12, 29, 50]. Microbial attack affects both the collagen and mineral phases of bone. Collagen is also lost via chemical hydrolysis [8, 29].

Additionally, bone molecular alterations have been assessed with infrared spectroscopy in archaeology [e.g. 7, 35, 51–54], forensic experiments [55, 56] and specifically for PMI estimations [56–58].

All the above-mentioned attempts to detect *in-situ* decomposition, ‘arrested putrefaction’, manner of death, mummification, post-mortem interval and other archaeological and forensic interpretations are underpinned by one unvalidated assumption: that putrefaction leads to bacterial degradation in bone and is caused by gut-bacteria. The latter can only be addressed by specific bone microbial studies, while the former can be answered by experimental studies with human remains. The only study that attempted to address this experimentally was undertaken by Mavroudas et al. [25] at a taphonomy facility in Texas, USA. They examined biodegradation in the rib, metatarsal and tibia from five human donors deposited in different settings (buried in soil, buried in a coffin, semi-buried in a coffin, surface deposition and exposed in a trench) for a period between 20 and 30 months. All bones showed very little bacterial histological modification. While these results are encouraging, the small sample size (only one donor per setting), methodological restriction to ground histology, and data collection completed by one observer demonstrates the need for a larger and more consistent experiment with human remains to confirm their observations and expand the study design by further methods such as scanning electron microscopy (SEM) and Fourier-transform infrared (FTIR) spectroscopy.

This experimental study sets out to test the assumption that putrefaction during the early stages of decay leads to bacterial degradation at the bone histological level. The aim is to study microscopic bone diagenetic changes in human remains, alongside pigs as non-human mammal samples, that have undergone putrefaction and decomposed while deposited on the surface in an experimental, controlled setting. Two methodological approaches are applied: (1) rib and femoral bone samples are assessed using conventional histological methods, and (2) changes in micro-structural and biomolecular properties are examined using SEM and FTIR spectroscopy; testing the null hypothesis that bone bioerosion and biomolecular properties, do not correspond to putrefaction experienced during early stages of decomposition.

## Materials and methods

Seventeen human donors and six pigs were deposited during different seasons on the surface in a known Australian environment and left to decompose. All the remains underwent all stages of decomposition reaching skeletonisation.

### Field site

**Fig 1:**
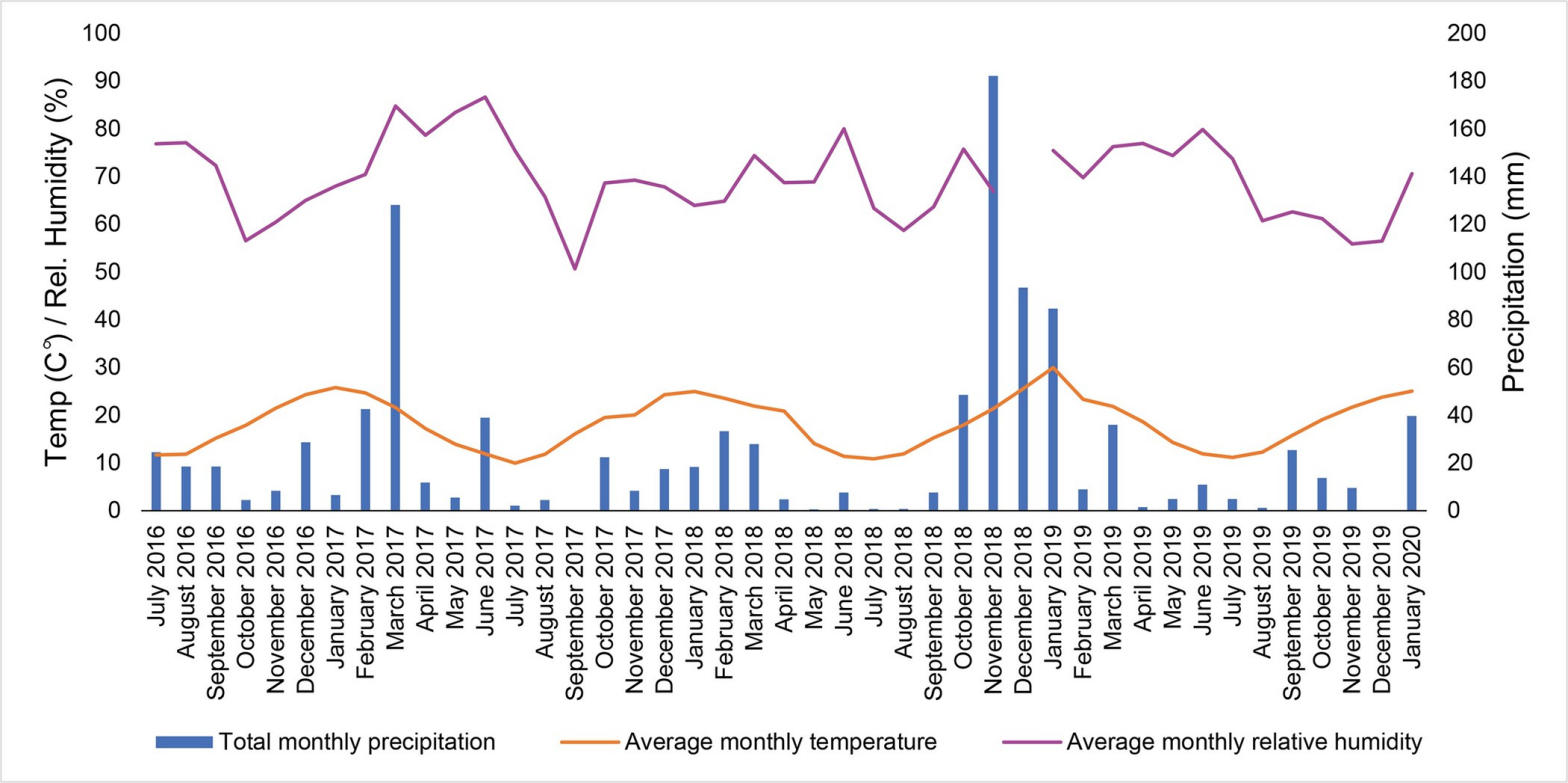
Average monthly temperatures, average monthly relative humidity and total rainfall readings during the study period. The gap in relative humidity is due to instrument failure.

The study was conducted at the Australian Facility for Taphonomic Experimental Research (AFTER), owned by the University of Technology Sydney (UTS) and established in collaboration with academic, police and forensic agencies. AFTER is a human decomposition facility dedicated to the study of forensic taphonomy where biological, physical and chemical processes of human decomposition are investigated under a variety of conditions. The facility is located in the Hawkesbury region of New South Wales in Australia, classified as climate zone 6 (mild temperate) by the Bureau of Meteorology. AFTER encompasses approximately 4.86 hectares of land and consists of an open eucalypt woodland, defined as Cumberland Dry Sclerophyll Forest [59]. The soil at the site is broadly classified as sandy clay loam or gravelly sandy clay with a yellowish to brown topsoil (Munsell code 10YR 5/3-5/4). The pH and electrical conductivity (EC) of topsoil at six different locations at the site measured an average of 5.2 and 80.9 μS/cm, respectively. Average monthly temperatures, average monthly humidity and total monthly rainfall are presented in Fig 1. While the study period ran from February 2016 to January 2020, the site’s weather station became operational at the end of June 2016.

### Human donors

The human cadavers used in this study were acquired through the Body Donation Program, overseen by the Surgical and Anatomical Science Facility (SASF) at the University of Technology Sydney. Consent was provided by the donor to use their remains for the purposes of research at AFTER, in accordance with the NSW Anatomy Act 1977. The study was approved by the UTS Human Research Ethics Committee (UTS HREC REF NO. 15–0029 and ETH18-2999), and the Science and Medical Delegated Ethics Review Committee at the Australian National University (ANU) (protocol 2019/041), and complies with all legal and ethical standards associated with the use of human remains for scientific research. All donor biodata were anonymised, and only basic medical history records were accessed to extract potential variables confounding the decomposition processes reflected in bone structure and histology.

In total, 17 individuals were selected by UTS to be destructively sampled. Donor details can be found in Table 1. The human sample comprised of 14 males and three females with age-at-death ranging from 59 to 93 years old. They were deposited between February 2016 and March 2018 and sampled on 25^th^ and 26^th^ June 2019.

**Table 1:** Donor and pig data.

Donor number	Sample number	Sample side (R = right or L = left)	Femur or Rib	Human (H) or Pig (P)	Season* of deposition	Days of deposition	Secondary placement (Yes or No)	Sex (M = male or F = female)	Age at death	Approx. Stature	Frozen (Yes or No) Not frozen = fresh	Clothing (Yes or No)	Position (S = supine, P = prone, L = laterial)
1602	HB03	R	Rib	H	Summer	1238	No	M	64	185	Yes	No	S
	HB04	R	Femur	H	Summer	1238	No	M	64	185	Yes	No	S
1603	HB05	L	Femur	H	Summer	1238	No	M	76	170	No	No	S
	HB06	R	Rib	H	Summer	1238	No	M	76	170	No	No	S
1617	HB07	R	Femur	H	Winter	1062	No	M	64	172	Yes	No	S
	HB08	R	Rib	H	Winter	1062	No	M	64	172	Yes	No	S
1618	HB09	R	Femur	H	Winter	1063	No	M	77	172	No	No	S
	HB10	R	Rib	H	Winter	1063	No	M	77	172	No	No	S
1619	HB11	L	Femur	H	Winter	1057	No	M	59	152	No	No	S
	HB12	R	Rib	H	Winter	1057	No	M	59	152	No	No	S
1705	HB13	R	Femur	H	Autumn	836	No	F	69	168	No	No	S
	HB14	R	Rib	H	Autumn	836	No	F	69	168	No	No	S
1621	HB15	R	Femur	H	Spring	949	No	M	77	171	No	No	S
	HB16	R	Rib	H	Spring	949	No	M	77	171	No	No	S
1716	HB17	R	Femur	H	Winter	725	No	M	91	167	No	No	S
	HB18	R	Rib	H	Winter	725	No	M	91	167	No	No	S
1722	HB19	L	Femur	H	Autumn	463	Yes	M	80	175	Yes	Yes	P
	HB20	L	Rib	H	Autumn	463	Yes	M	80	175	Yes	Yes	P
1718	HB21	R	Rib	H	Winter	717	No	M	74	176	No	No	S
	HB22	R	Femur	H	Winter	717	No	M	74	176	No	No	S
1717	HB23	R	Femur	H	Winter	718	No	F	67	167	No	No	S
	HB24	R	Rib	H	Winter	718	No	F	67	167	No	No	S
1721	HB25	R	Femur	H	Autumn	463	Yes	M	74	180	Yes	Yes	S
	HB26	R	Rib	H	Autumn	463	Yes	M	74	180	Yes	Yes	S
1805	HB27	R	Femur	H	Autumn	463	Yes	F	93	156	Yes	Yes	S
	HB28	R	Rib	H	Autumn	463	Yes	F	93	156	Yes	Yes	S
1806	HB29	R	Femur	H	Autumn	463	Yes	M	73	164	Yes	Yes	S
	HB30	R	Rib	H	Autumn	463	Yes	M	73	164	Yes	Yes	S
1706	HB33	R	Femur	H	Autumn	830	No	M	74	180	No	No	S
	HB34	R	Rib	H	Autumn	830	No	M	74	180	No	No	S
1719	HB35	R	Femur	H	Winter	714	No	M	89	163	No	No	S
	HB36	R	Rib	H	Winter	714	No	M	89	163	No	No	S
1803	HB37	R	Femur	H	Summer	531	No	M	87	172	No	No	S
	HB38	R	Rib	H	Summer	531	No	M	87	172	No	No	S
4041	PB40	R	Femur	P	Summer	583	No	*Not applicable*			No	No	L
	PB41	R	Rib	P	Summer	583	No	*Not applicable*			No	No	L
4243	PB42	R	Femur	P	Spring	83	No	*Not applicable*			No	No	L
	PB43	R	Rib	P	Spring	83	No	*Not applicable*			No	No	L
4446	PB44	R	Femur	P	Spring	78	No	*Not applicable*			No	No	L
	PB46	R	Rib	P	Spring	78	No	*Not applicable*			No	No	L
4849	PB48	R	Femur	P	Autumn	268	No	*Not applicable*			No	No	L
	PB49	R	Rib	P	Autumn	268	No	*Not applicable*			No	No	L
5051	PB50	R	Femur	P	Winter	237	No	*Not applicable*			No	No	L
	PB51	R	Rib	P	Winter	237	No	*Not applicable*			No	No	L
3902	PB02	R	Femur	P	Autumn	390	No	*Not applicable*			No	No	L
	PB39	R	Rib	P	Autumn	390	No	*Not applicable*			No	No	L

*Note that in Australia the seasons change on the first of September, March, June and December rather than the 21^st^.

**Fig 2:**
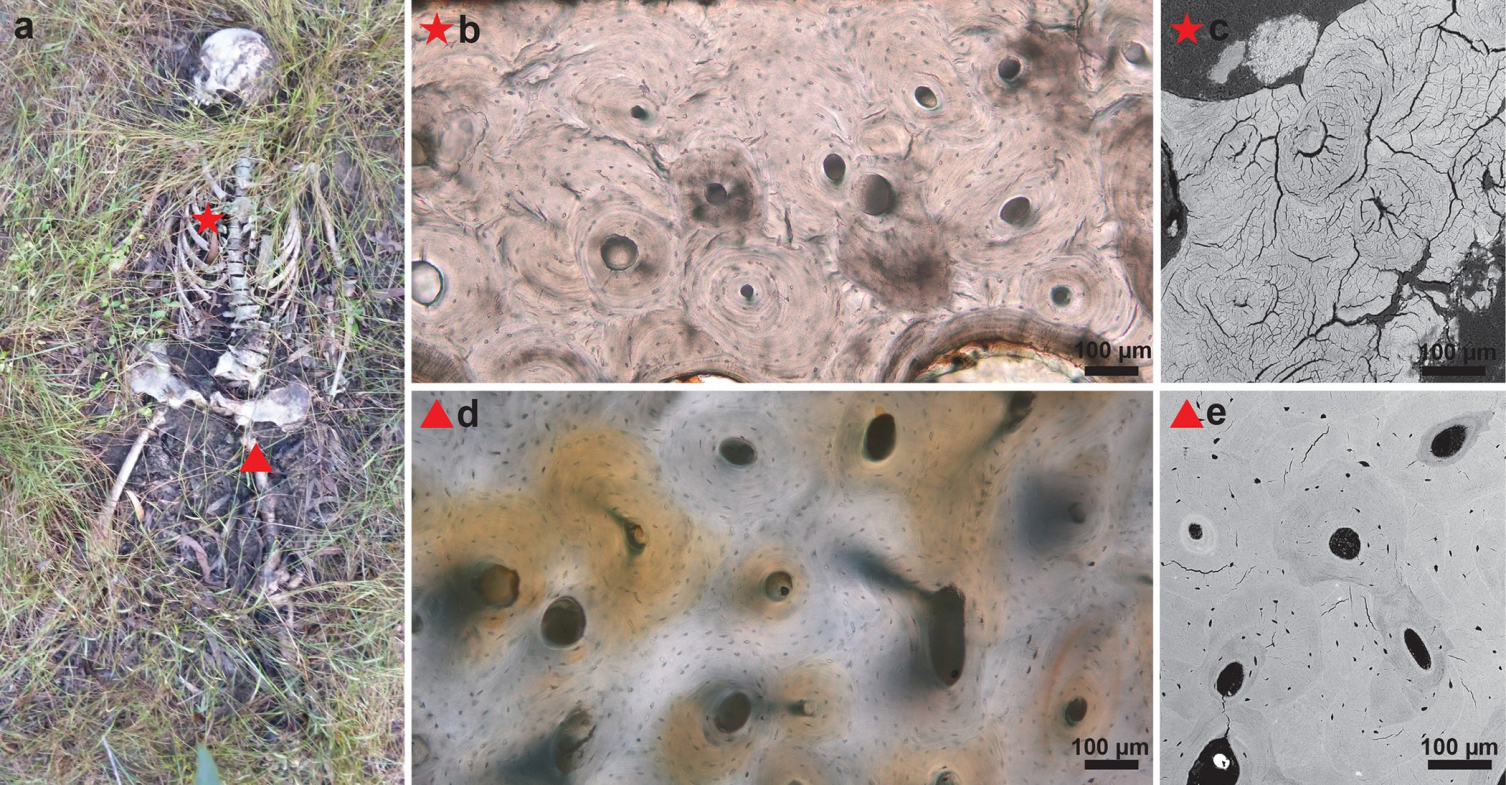
(a) After 1057 days of deposition, a rib and femoral sample were taken from donor 1619 which was placed in a supine, extended position. The red star and triangle indicate the sample locations; (b) Rib histological section showing the onset of degradation; (c) SEM image of the same rib section reveals the presence of several microcracks; (d) Histological section of the femur shows well preserved bone with clear osteocyte lacunae. The orange discolouration indicates former blood supply; (e) SEM image of the same femoral section with only a few microcracks present.

**Fig 3:**
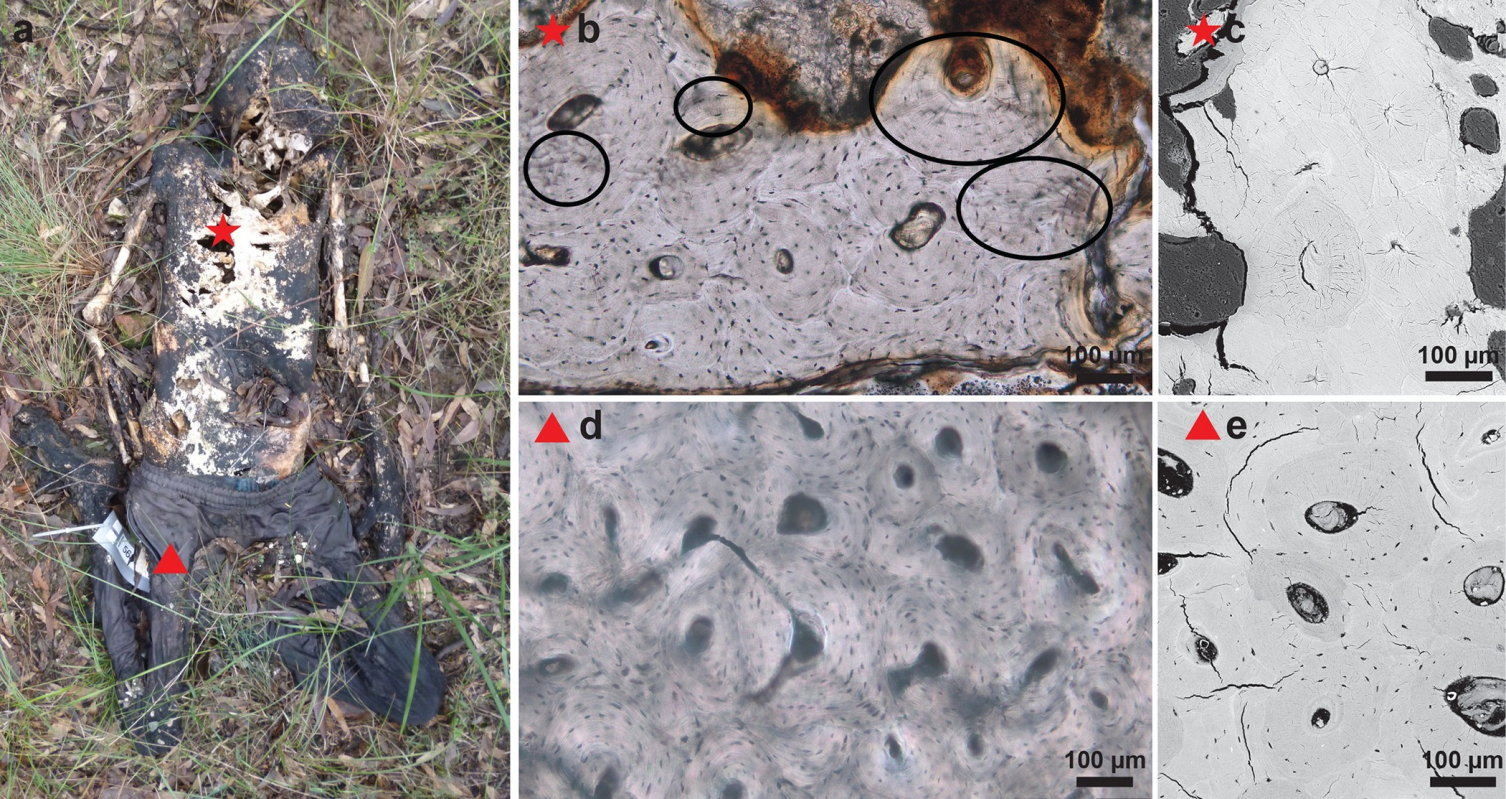
(a) Donor 1721 was frozen before being placed at AFTER. Rib and femoral samples were collected after 463 days of deposition. The clothed subject was deposited in a supine position with flexed lower limbs. The red star and triangle indicate the sample locations; (b) Rib histological section showing the onset of degradation; (c) SEM image of the same rib section reveals a limited number of microcracks; (d) Histological section of the femur showing well preserved bone; (e) SEM image of the same femoral section with a few microcracks.

Eleven individuals were control replicates (fresh, supine, unclothed; Fig 2) deposited in different seasons (Summer N = 2; Autumn N = 2; Winter N = 6; Spring N = 1). The remaining six donors were variation experiments that had been frozen prior to deposition in summer (N = 1), autumn (N = 4) and winter (N = 1). Four of these had been moved to a secondary location as part of another experiment (1721, 1722, 1805 and 1806), of which one was placed in a prone position and clothed (1722). The three other clothed individuals were deposited supinely with loosely flexed lower limbs (1721, 1805, 1806) (Table 1, Fig 3). Images of each donor at the time of sampling can be found in S1 Table.

Non-frozen donors (N = 11) were stored at 4°C for maximum 48 h prior to placement at AFTER. Frozen donors (N = 6) had been stored at UTS at -28°C. All donors were placed on the surface in separate plots measuring 5 x 5 m^2^, covered with a metal cage to prevent animal scavenging. Time-since-deposition ranged from 1238 days (3.3 years) to 463 days (1.2 years). All donors progressed through distinct stages of decomposition: fresh, bloat, active decay, advanced decomposition and skeletonisation, which took a minimum of one year. Some donors still had dried remnants of skin (e.g. donors 1721, 1722, 1803, 1805, 1806; see S1 Table), but all of them were classified as being in the skeletonisation state following Megyesi et al.’s [60] description. Thus, every donor underwent putrefaction before being sampled.

### Pigs

Six domestic pigs (*Sus scrofa)* were purchased post-mortem from a licensed abattoir, therefore requiring no ethics approval in accordance with the Australian Code of Practice for the Care and Use of Animals for Scientific Purposes (2004). The pigs were killed by a captive head bolt and transported to AFTER within 1 hour of death between January 2018 and November 2019. The pigs were placed on their side (laterally) outside the AFTER facility to comply with licensing agreements. Because the experiments were part of another project [61], they were not all placed on the same side. Two pigs were placed on the right side (pigs 4041, 4243) and four pigs were placed on their left side (pigs 4446, 4849, 5051, 3902). They were deposited a minimum of 20 m apart from one another and a minimum of 100 m away from the human cadavers. All pigs were covered with a metal cage to prevent animal scavenging, and protected with metal mesh once the experiment finished. However, one pig became scattered by a monitor lizard, known as goanna *(Varanus)* (pig 4446).

Similar to the human cadavers, the pigs were deposited in different seasons (Summer N = 1; Autumn N = 2; Winter N = 1; Spring N = 2). Time-since-deposition ranged from 583 days (1.5 years) to 78 days (2.5 months) (Table 1). Sampling took place in June 2019 and January 2020. All pigs progressed through distinct stages of decomposition: fresh, bloat, active decay, advanced decomposition and skeletonisation, although three of them had still remnants of dry skin and fur (pigs 4243, 4849, 3902), similar to some of the humans. Bone samples were collected from rib and femur in the skeletonisation stage between June 2019 and January 2020. Images of each subject at the time of sampling can be found in S1 Table.

### Bone sampling

For the human donors as well as the pigs, the right femur and right 3^rd^ rib were targeted in this study in order to: 1) account for intra-individual variation in diagenesis, and 2) validate if the rib is more affected by internal bacteria because of its ‘closer’ location to the gut [32, 33]. In three human cases the left femur had to be sampled because of the presence of a hip joint implant on the right side (samples HB05, HB11, HB19). In one case (sample HB20), the left rib was sampled because of access reasons (donor 1722 was placed in a prone position wearing clothes).

**Fig 4:**
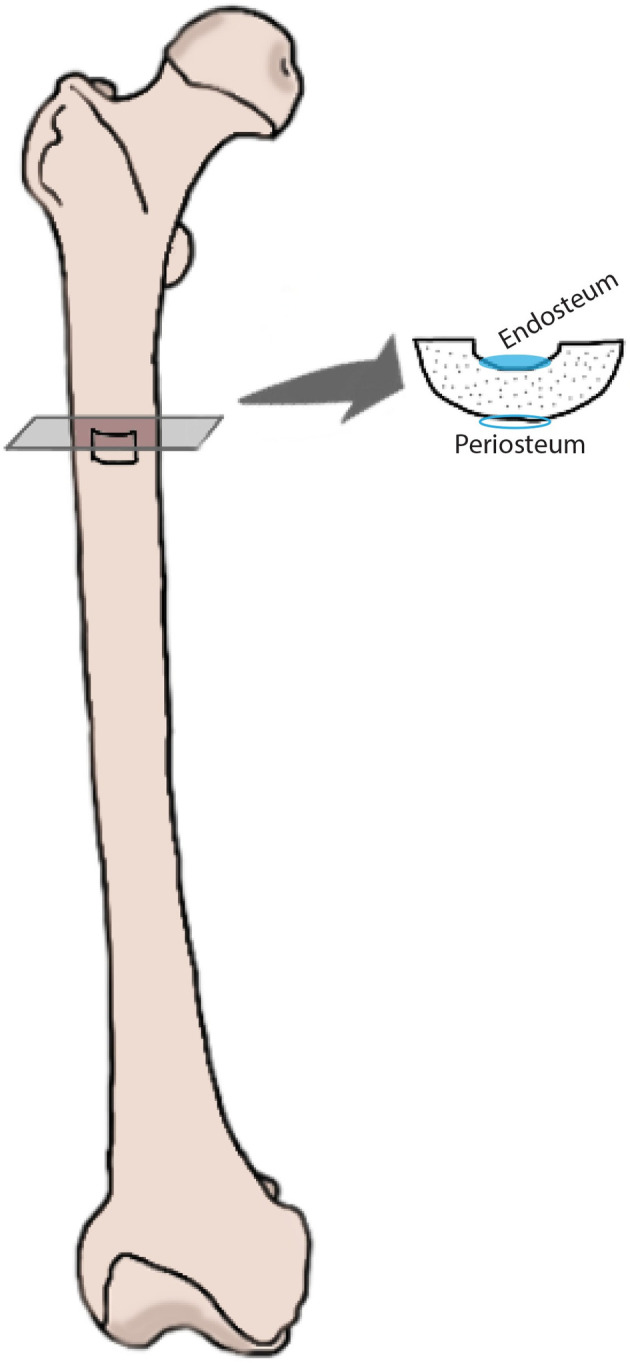
Femur samples were taken from the anterior side of the bone diaphysis, approximately 4–5 cm from the lesser trochanter. The endosteal and periosteal surfaces were scraped for analysis using FTIR spectroscopy.

**Fig 5:**
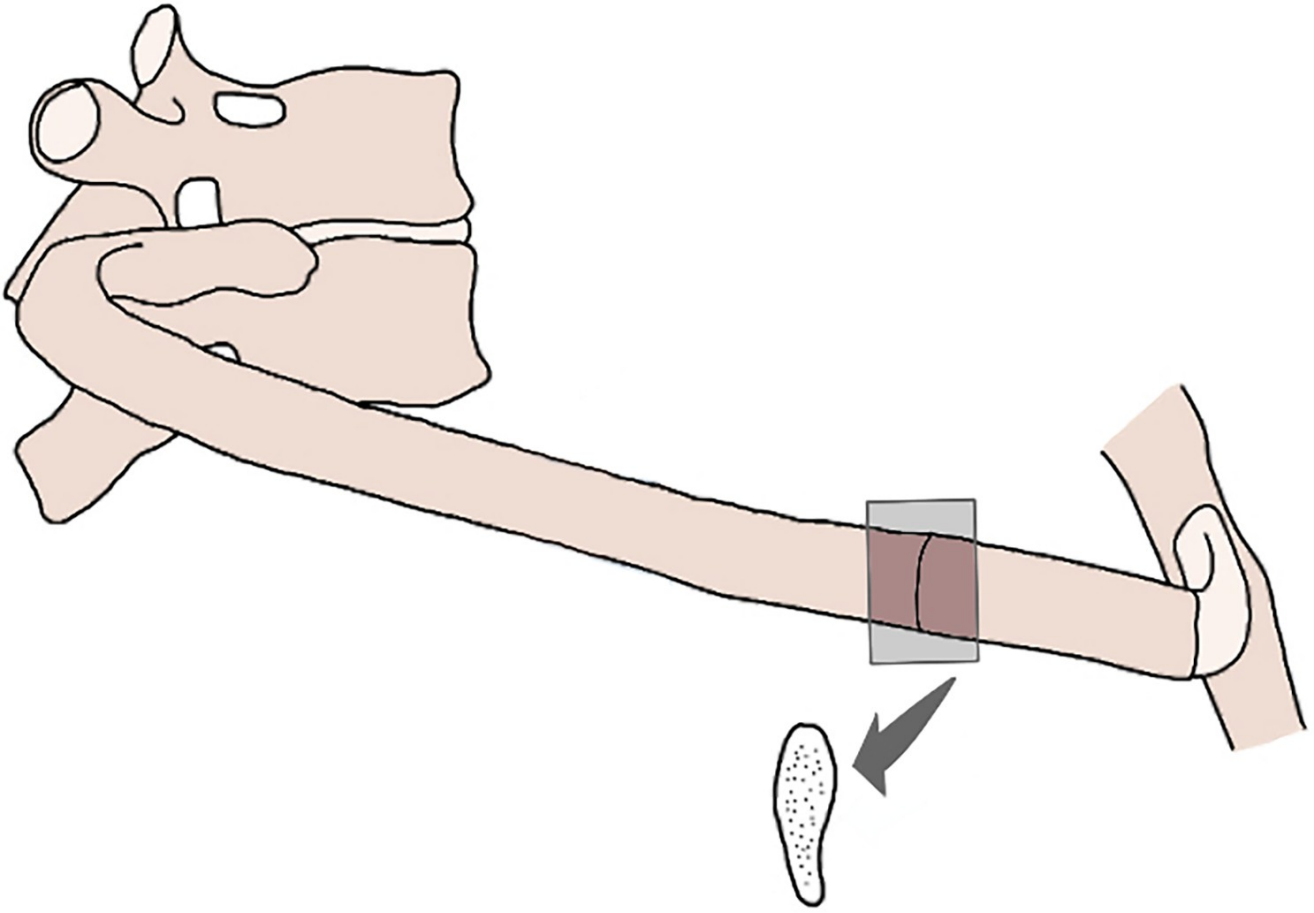
The 3^rd^ right rib was sampled 2–3 cm from the sternal end.

A cross-section at approximately 4–5 cm down from the lesser trochanter was made with a Ryobi (R18MT Type II) Multitool with oscillating saw blade. A full cross-section was taken because it had been designated for another project at AFTER [62]. A bone sample of 1 cm^2^ was sub-sampled at the anterior side with a Dremel 8220 multitool with Lock Mandrel bit (Dremel EZ402) and diamond cutting wheel (Fig 4). The same tool was used to cross-cut the 3^rd^ right rib at 2–3 cm from the sternal end (Fig 5). All bone samples were immediately frozen and kept in the freezer until processed for this study.

### FTIR spectroscopy

FTIR spectroscopy was performed on powder scrapings of the endosteal and periosteal surface of all femoral samples to examine biomolecular changes inside the medullary cavity and at the outer surface of the bone (Fig 4). The rib samples were not targeted because of their thin cortical bone and the fact that the endosteal surface was not accessible without cutting the rib transversally. Bone scrapings were manually ground using an agate pestle and mortar. About 1 mg of bone powder of 20–50 μg particle size was analysed in triplicate with a Bruker Alpha with a diamond attenuated total reflectance (ATR) crystal. The crystal was cleaned with ethanol between every sample measurement. A background measurement was run before each sample measurement. A total of 64 scans were performed for all measurements with a resolution of 4 cm^-1^ in the 400–4000 cm^-1^ spectral region at room temperature of 20°C.

Spectral analysis was performed using the OPUS 7.5 software (Bruker). Data processing was performed in the 400–1800 region. Spectra were normalised to the phosphate peak near 1030 cm^-1^ for comparison. The following FTIR indices were calculated: (1) Mineral changes were analysed by measuring the crystallinity index (CI) or infrared splitting factor determined based on the phosphate band splitting in the 700 to 500 cm^-1^ region of the spectrum according to Weiner and Bar Yosef [54]:

$$CI=\frac{I_{561}+I_{601}}{I_{587}}$$

where *I*_561_ is the intensity of the peak at 561 cm^-1^, *I*_601_ is the intensity of the peak at 601 cm^-1^ and *I*_587_ is the intensity at the trough between the 561 cm^-1^ and 601 cm^-1^ peaks. (2) Over time hydroxyapatite becomes more ordered and carbonate decreases in the inorganic phase. The carbonate to phosphate ratio (C/P) was determined from the intensity of the carbonate and phosphate modes at 1415 (CO_3_^2-^) and 1030 cm^-1^ (PO_4_^3-^), respectively. This measurement can be interfered with by the presence of collagen and is thus only used as a semi-quantitative measure [54, 63]. (3) Similarly, the organic content of the bone was measured using the amide to phosphate ratio, based on the maximum absorbance of the amide I band at 1645 cm^-1^ to that of the phosphate band at 1030 cm^-1^ [64]. The determination of the CI, and the carbonate to phosphate and the amide to phosphate ratios was based on the average values from the spectra of three individual samples from each specimen. The standard deviation for the CI was circa 2%, while for the carbonate to phosphate and the amide to phosphate ratios was the standard deviation was circa 7%.

### Histology

#### Preparation procedures

The bone samples were processed into thin sections following standard methods [e.g. 65, 66]. All preparation was conducted under the same laboratory conditions following the technical protocol of the Hard Tissue Histology Laboratory of the ANU School of Archaeology and Anthropology. Samples were embedded in Buehler EpoxiCure^TM^ epoxy resin (4 to 1 ratio of resin and hardener, respectively) inside dedicated plastic moulds with removable lids. Each embedded block was next cut on a low-speed high precision diamond saw (Kemet Micracut® 151) to reveal two transverse histology surfaces of cortical bone. Both revealed surfaces were polished on a high grit (grit 600/P1200) pad to remove any scratches created by the rotating blade. One surface was used for SEM analysis. The other surface was mounted onto a glass slide using Stuk epoxy glue. Once set on the glass slides, the samples were trimmed on the same saw to down 500 μm sections. These were next ground down on a series of grinding pads, beginning at a coarse grit (grit 320/P400) and gradually progressing to a finer grit (grit 600/P1200). Once a thickness of approximately 100–120 μm was reached, the ground slides were polished using a Buehler diamond polishing paste (Buehler Micropolish II 0.3 μm powder). The thin sections were regularly checked under the microscope to ensure clear visibility of all histological features. The ground and polished slides were then placed in an ultrasonic bath cleaner to remove any debris that could have entered from the technical preparation steps. They were next dehydrated in a series of ethanol baths, cleared in xylene and covered with a glass slip mounted using dibutylphthalate polystyrene xylene (DPX).

#### Analysis of the histology sections with light microscopy

The thin sections were examined using standard high powered light optical microscopy. Two observers (ES and JM) viewed slides independently at the University of Wollongong and the Australian National University. Respectively, an Olympus BX51 microscope equipped with a DP23/DP28 high resolution camera and a BX53 microscope with a DP74 camera were used, both operated by cellSens Olympus (Evident) software. Each thin section was imaged in its entirety under transmitted and linearly polarised light under x100 magnification. The scans of the entire thin sections were automatically assembled at magnification of x100 using the cellSens instant microscope image analysis tool which stiches neighbouring images in live scanning mode.

In this study, histotaphonomic alterations were first analysed based on the two most important indications of biodegradation: preservation of microstructure and birefringence:

1. The percentage of intact bone microstructure was scored using the OHI [17, 18]. This index, used in archaeology, is based on a visual examination of histological features assigning an ordinal score of six ranging from 0 (<5%) poorly preserved and only Haversian canals identifiable with most bone matrix being destroyed; 1 (< 15%) small amounts of bone can be identified but the remainder of bone matrix is destroyed with evidence of microscopical focal destruction (MFD); 2 (<50%) larger patches of bone preserved amongst largely destroyed bone matrix; 3 (>50%) areas of bone are well-preserved such that more than half of the sample is well-preserved and the remainder destroyed; 4 (>85%) only small patches of bone are poorly preserved; and 5 (>95%) almost the entirety of the sample is well-preserved and bone is comparable to samples from modern cadavers.
  The type of bacterial MFDs was not described in this study. In the past, different MFDs have been categorised based on their morphology into linear longitudinal, budded, lamellate destruction [13] and Wedl tunnels which are meandering MFDs originally attributed to the action of fungi [10, 21, 67, 68] but recently found to be caused by cyanobacteria [8, 11]. While originally considered in this study, the morphological description of MFDs appeared restricting, inconsistent and subjective. Moreover, not the shape but the presence of MFDs was the focus of this study.
2. Collagen birefringence can be assessed in bone because of its optical anisotropy such that when polarised light is shone, the orientation of collagen fibres will be reflected in dark and/or light colouration. In bone, histologists typically look for dark, light and alternating micro-morphologies, called morpho-types in individual osteons [69] which indicate longitudinal, transverse and angled (alternating) orientation of collagen fibres [69, 70]. The intensity of birefringence is dependent on the quantity and orientation of the collagen fibres, the presence of the bone mineral and the orientation of the section. The degree of birefringence can thus be useful to indicate to what extent collagen has degraded. In the archaeological literature there appears to be a lack of standardisation in the way scores are assigned and defined with Brönniman et al [15] using a scale from 1 to 5 with vague descriptions and, in contrast to the OHI, no use of score 0. Another scoring system was created by Caruso et al. [71] where birefringence was assessed as high, medium or low intensity. After comparison, and for the ease of replication, this study used the scoring introduced by Jans et al. [19] with the intensity of birefringence represented by 1 = normal and comparable to fresh bone, 0.5 = reduced birefringence and 0 = absent birefringence. Due to the absence of cracks under the microscope, cracking levels were not scored.

The histological images with magnification of x100 were analysed independently by two observers (ES, JM) and scored in triplicate with 4 weeks and 1 year apart (S2 Table). In addition, a more detailed examination under the microscope took place using other magnifications (x40, x100, x200) so that every section could be described individually.

To assess potential associations between the histological observations and the depositional variables (human *vs* pig, femur *vs* rib, fresh *vs* frozen, primary *vs* secondary, season, position, time-since-deposition), the following statistical approach was employed using statistical software Stata 17 (S1 File). The pig-only sample was too small for reliable statistical analysis (N = 12). The human-only sample was larger (N = 34) but lacked variability in the scores. Furthermore, the results for both groups were skewed (>74% of the samples were either OHI 5 or CB 1) (Tables 2 and 3). For these reasons the scores were recoded as binary variables: (OHI 5 and <5; CB 1 and <1) based on the argument that they were data driven. Variables for the total sample (humans and pigs) were used for statistical analysis. Fisher’s exact tests were used for categorical relationships. For days of deposition as continuous predictor variables, logistic regression with a cluster correction (two measures per individual) was used to control the standard error due to repeated measures. The correlation between OHI and CB scores was calculated using Kendall’s Correlation, used to measure the correlation between ordinal variables when the sample size is small and when many similar scores are obtained.

**Table 2:** Median OHI (left) and CB (right) scores of thin sections of human femora and ribs. Each specimen was assessed by two observers, with every observer scoring three times.

Humans (N = 34)					
OHI (median)			CB (median)		
Scoring	N	%	Scoring	N	%
**5**	34	100	**1**	34	100
**4**	0	0	**0.5**	0	0
**3**	0	0	**0**	0	0
**2.5**	0	0			
**2**	0	0			
**1**	0	0			
**0**	0	0			

**Table 3:** Median OHI (left) and CB (right) scores of thin sections of pig femora and ribs. Each specimen was assessed by two observers, with every observer scoring three times.

Pigs (N = 12)					
OHI (median)			CB (median)		
Scoring	N	%	Scoring	N	%
**5**	0	0	**1**	1	76.09
**4**	4	33.33	**0.5**	9	19.57
**3**	5	41.67	**0**	2	4.35
**2.5**	1	8.33			
**2**	2	16.67			
**1**	0	0			
**0**	0	0			

Inter-rater agreement was calculated with Cohen’s Kappa. Intra-rater agreement was assessed using the interclass correlation coefficient (ICC).

#### Analysis of the histology sections with scanning electron microscopy

The polished surface of the embedded blocks was examined with a Phenom XL scanning electron microscope with Phenom XL silicon drift detector in low vacuum mode. Backscattered electron images were taken with a beam accelerating voltage of 15kV. Energy-dispersive spectroscopy (EDS) analyses were carried out with PhenomProSuite software. Backscattered SEM is well suited for studying different types of bio-erosion in bone because diagenetic microstructural and chemical changes can be studied at a smaller resolution in combination with elemental analysis [8, 72]. Bacterial attack is responsible the enzymatic digestion of collagen and the initial dissolution of hydroxyapatite, while the local hydrological conditions will determine where the mineral will reprecipitate [30]. Dissolution appears on SEM images as grey/black (less dense) areas and remineralisation will come up as bright/white (dense) areas. In addition, physical degradation such as microcracking and bacterial tunnels are visualised and any inclusions such as fungi, roots or other minerals can be identified.

## Results

### Histology results

The agreement between the two raters was almost perfect (Kappa 0.8). The reliability per single rater was excellent (ICC = 0.94) and good (ICC = 0.84). Images of the subjects, the entire histological sections and SEM at x350 magnification can be found in S1 Table, statistical details can be found in S1 File.

Upon visual examination of the sections, the general tenor was that the human sections appeared well preserved with low levels of bacterial attack and low levels of collagen degradation (Figs 2 and 3). SEM analysis confirmed the well-preserved state of the sections with absence of de- and hypermineralised areas and inclusions. The sections showed absent or very limited cracking when observed with light microscopy, however microfissures were observed in all sections with SEM. Ribs clearly had more microcracks than femoral sections except for some the most recently placed donors (1719, 1721, 1803, 1805, 1806) (Fig 2 and S1 Table).

**Fig 6:**
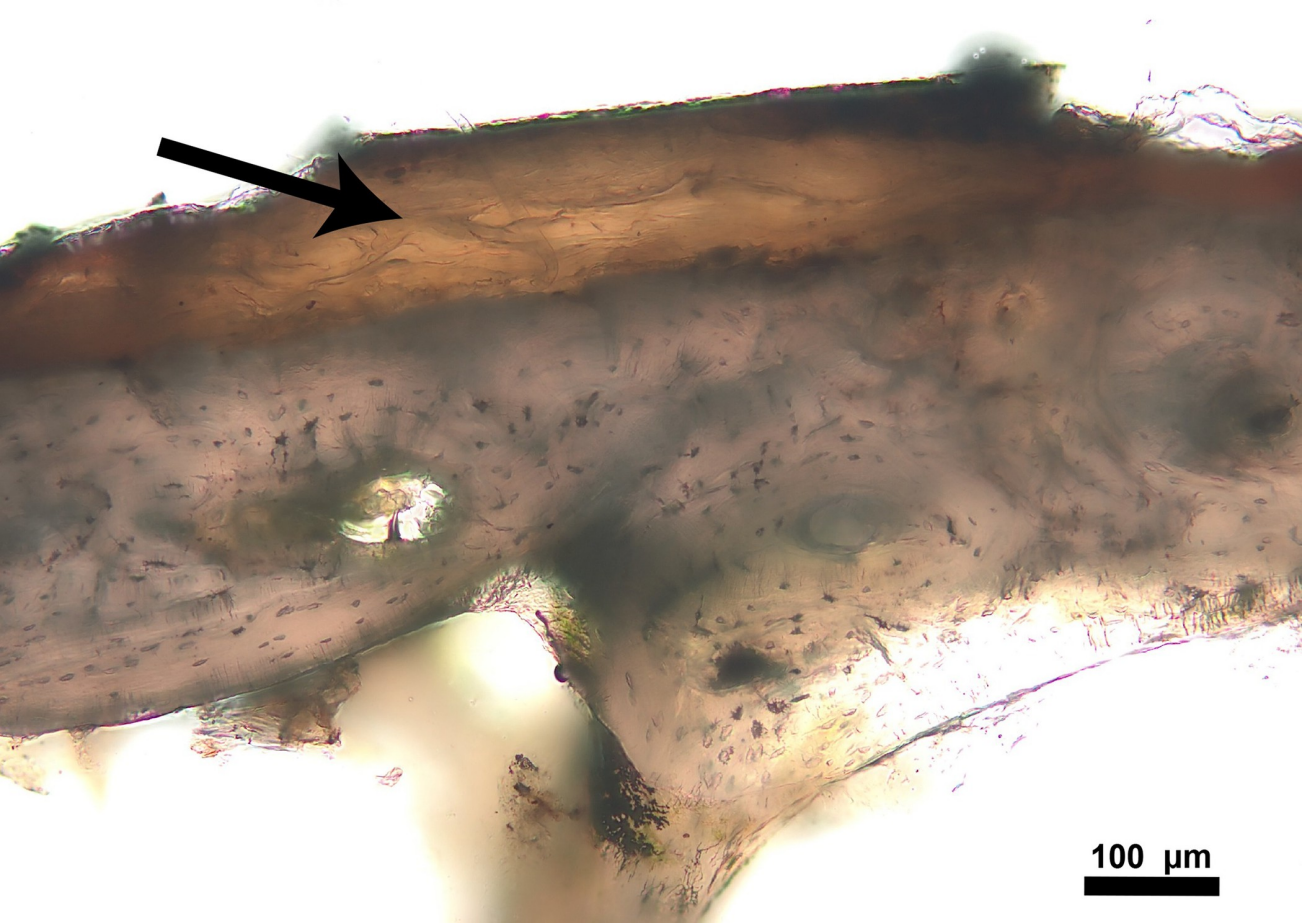
Rib thin section of donor 1618 with periosteum still present (arrow).

**Fig 7:**
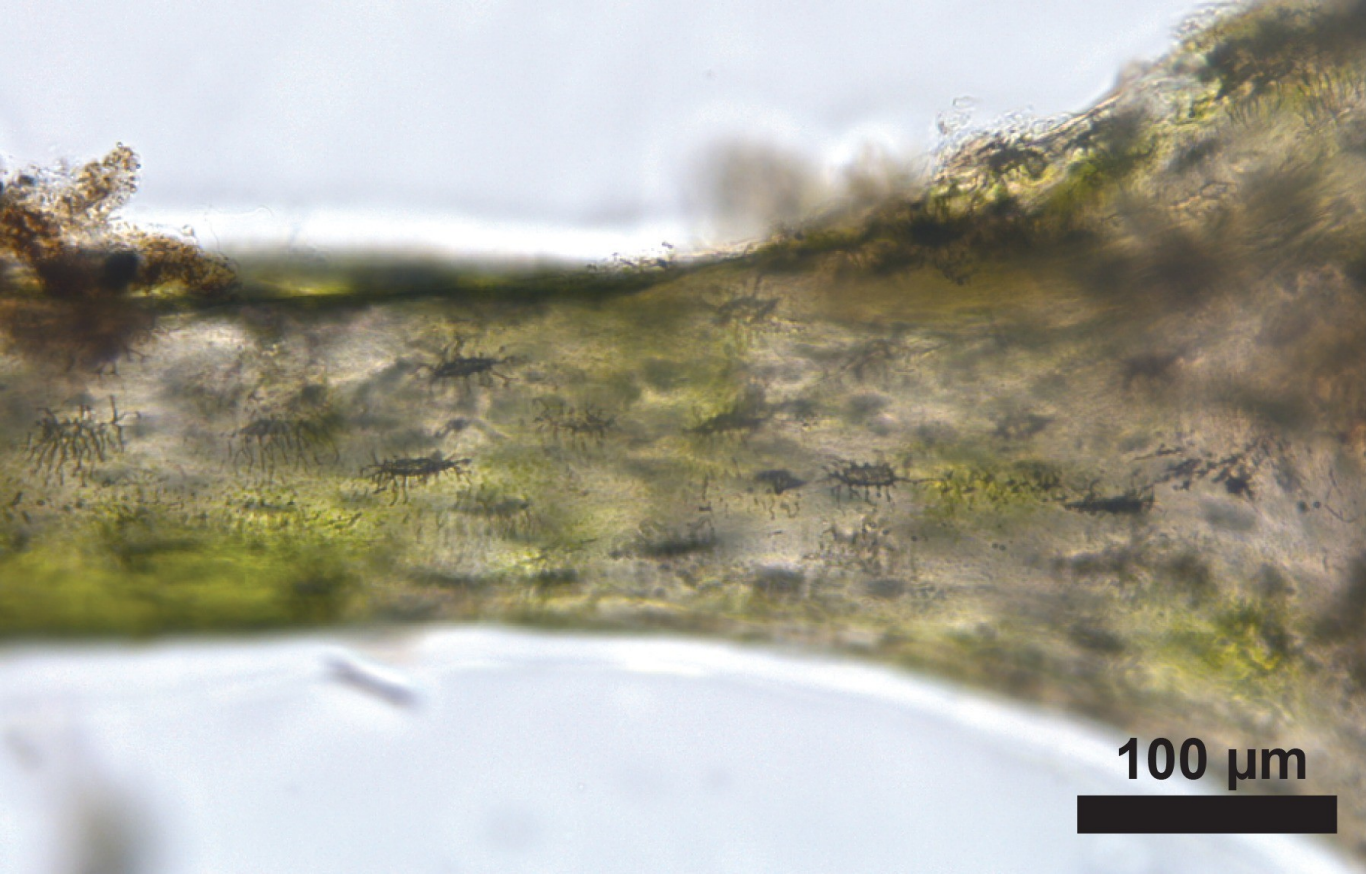
Z-stack of rib thin section of donor 1618 with the presence of moss displayed as green patches.

**Fig 8:**
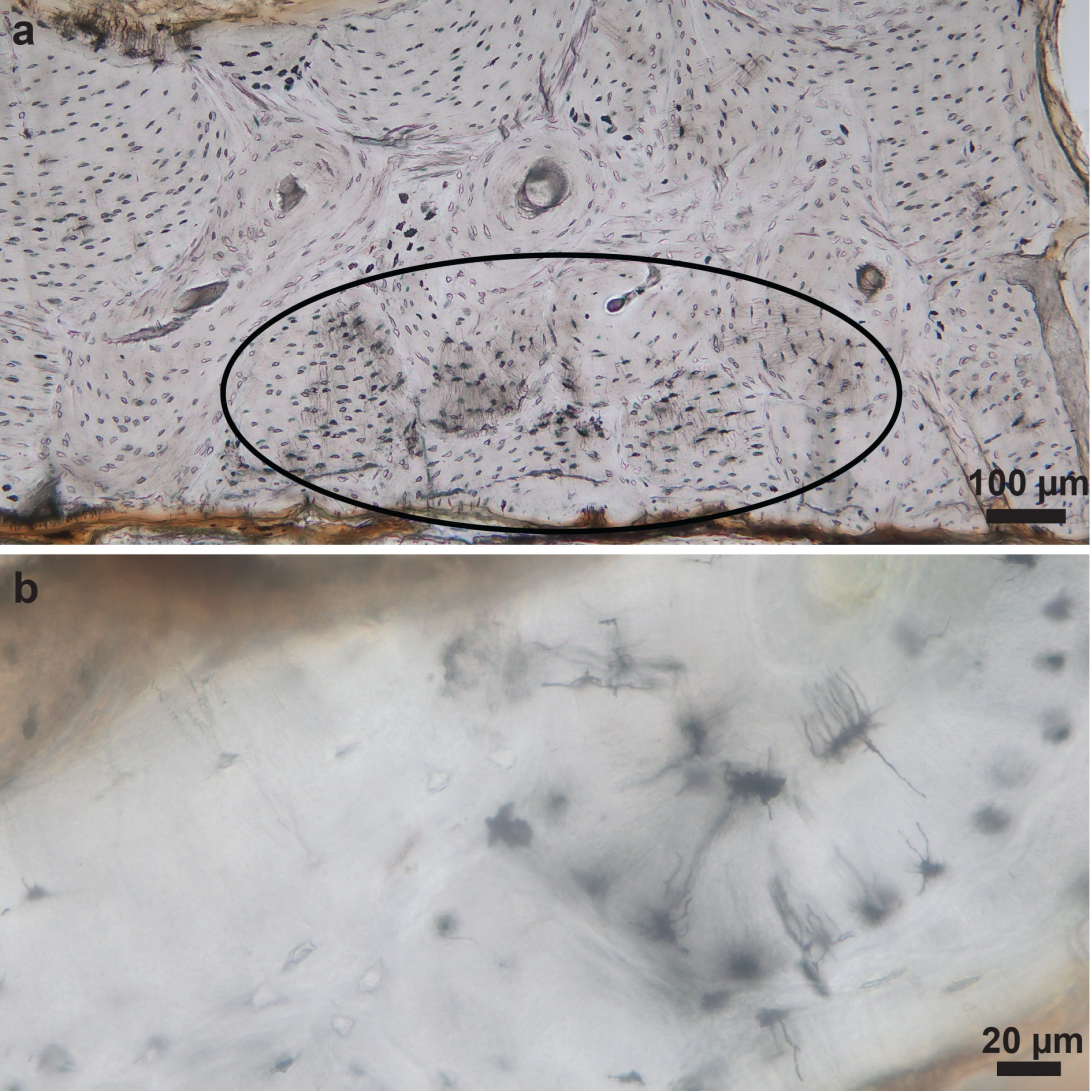
Rib thin sections of donors 1717 (a) and 1705 (b) showing the onset of degradation.

While macroscopically the bones appeared in a dry state, 20 out of 34 human sections contained yellow patches of degrading soft tissue, either as discolouration from former blood supply (Fig 2D) or in the presence of periosteum (Figs 3B and 6). Five human sections contained green discolouration’s assumed to be moss growth (Fig 7). Upon higher magnification (x200), all human ribs (N = 17) showed the onset of biodegradation (Figs 2B, 3B, 8A and 8B), which did not cover more than 5% of the sample, hence still scored OHI 5. For femurs this was the case in 14 out of 17 sections.

**Fig 9:**
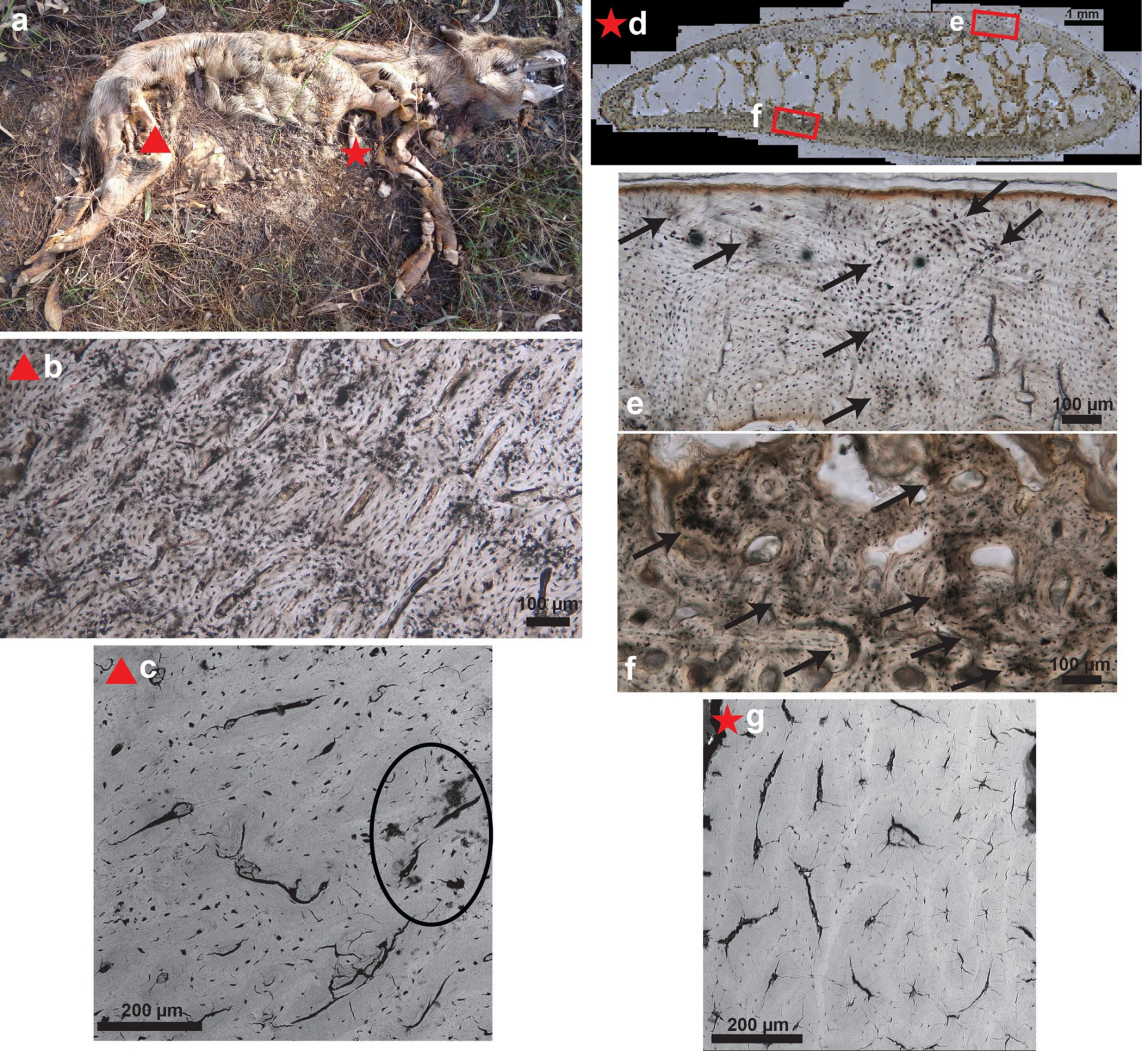
(a) Pig 3902 was deposited on the left side for 390 days. The red star and triangle indicate the sample locations; Both (b) femur and (d) rib thin sections displayed degradation; The rib section displayed more degradation on (f) the side that was in contact with soil, than on (e) the uppermost side; SEM images show (c, g) limited microfissures and (c) the onset of demineralisation in the femoral sample (circled).

Regarding the pig sections, soft tissue was also present (8/12) but moss was not observed (N = 0). Overall, there was more degradation in the pig sections than in the human thin sections with the rib sections slightly worse preserved than the femoral sections and the onset of demineralisation as observed with SEM (Fig 9C, 9D and S1 Table). The rib sections displayed more MFD on the sides that were in contact with the soil (Fig 9D–9F) while patterns of MFD were less clear in the femoral sections.

Tables 2 and 3 summarise the median OHI and CB scores of each of the human and pig samples (both femora and ribs).

Regarding OHI, a strong statistically significant relationship was noted between the scores of pigs and humans (*p*<0.0005). All the pig samples received a median OHI score less than 5, while all the human samples had a median overall score of 5.

There was no statistically significant correlation between the OHI scores of femora and ribs (*p* = 1). This is most likely related to the fact that all pigs received a OHI median score of less than 5, independently of femur and rib. Similarly, there was no statistically significant relationship between the different seasons of deposition (*p* = 0.107), and no significant relationship between primary or secondary depositions (*p* = 0.90). There appeared to be a statistically significant correlation between the OHI scores in fresh and frozen individuals (*p* = 0.02) although it is important to note that all <5 scores were corresponding to pigs that were not frozen. Looking at the human data only, there was no variability between the fresh and frozen human data, all scoring 5. Finally, there was no statistically significant relationship between the OHI scorings and days of deposition (*p* = 0.08).

The results of the collagen birefringence (CB) scoring gave similar results. A strong statistically significant correlation was observed in CB scores between humans and pigs (*p*<0.0005). No humans received a CB that was less than 1. There was no significant correlation between femur and rib (*p* = 1), neither with season of deposition (*p* = 0.075), primary or secondary placement (*p* = 0.09) and days of deposition (*p* = 0.12). Similar to OHI scores, a statistically significant relationship was detected between frozen and fresh subjects (*p* = 0.044). However, it is important to note that 11 out of 12 of the <1 scores were fresh, including the lower scorings of pig sections.

Finally, a statistically significant correlation was identified between median OHI and CB scores (*p*<0.0005).

### Biomolecular results

**Fig 10:**
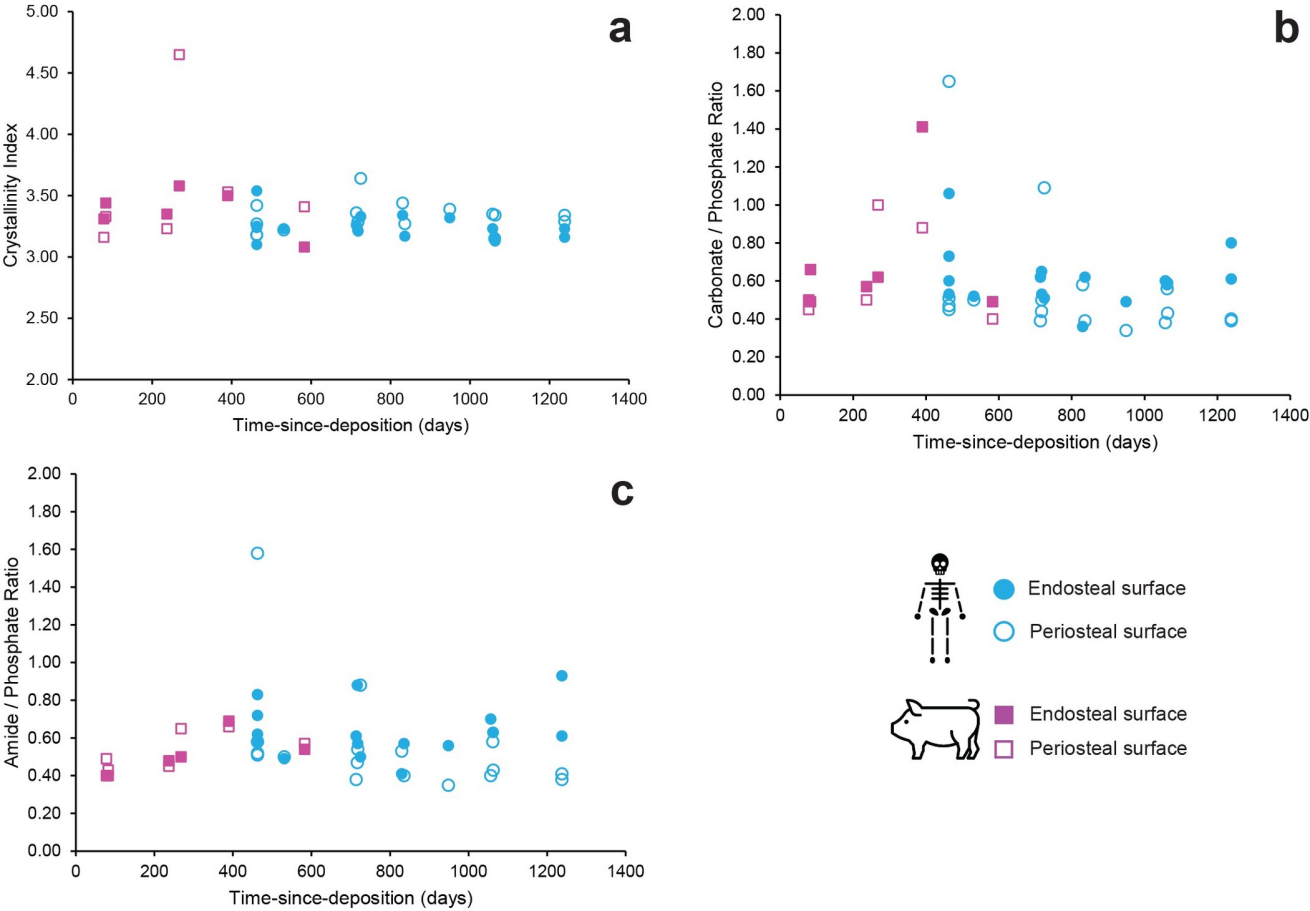
(a) Crystallinity index; (b) carbonate to phosphate ratio; and (c) organic content at the endosteal (inner) and periosteal (outer) surface of the femur samples from both human and pig subjects.

Infrared spectroscopic analysis of the human and pig bones revealed no trends for the investigated time-since-deposition between 463 and 1238 days with linear correlation coefficients (R^2^) in the range 0.01 to 0.3 as is evident from the data plotted in Fig 10 (also see S1 File). Fig 10A illustrates the crystallinity index for the bones as a function of time-since-deposition and shows that there is no increase in crystallinity over time at the inside (endosteal surface), as well as the outside (periosteal surface) of the femoral bone samples during the experimental period. The carbonate to phosphate ratio shows the same result, indicating that hydroxyapatite did not become more ordered and carbonate levels did not decrease during the experimental period (Fig 10B). The organic content of the bone, measured by the ratio of amide I to phosphate, did not indicate any changes in humans as well as pigs (Fig 10C).

## Discussion

This study confirms the null hypothesis that histological markers of bioerosion do not correspond to putrefaction experienced during the early stages of human decomposition. All humans in this study underwent all stages of decay, including putrefaction. They displayed well-preserved bone on a microscopic histological level with unaltered molecular composition. The results have implications for investigations into early post-mortem interval in forensic anthropology and the interpretation of early post-mortem practices in archaeology. This key finding contradicts the assumption that bioerosion reflects the extent to which remains are exposed to putrefaction [9, 15, 23, 38]. Thus, using the presence of MFD in bone as an argument to prove putrefaction, *in-situ* decomposition, early taphonomic processes or use it as argument for primary burial [e.g. 15, 42, 43] cannot be supported based on the experimental human data presented here. These results are in agreement with experimental data obtained by Mavroudas et al. [25] in their Texas experiment on five donors placed in different burial scenarios.

There was no statistically significant correlation between the OHI scores of femoral and rib samples. Visually, many samples with an OHI 5 appeared to have the onset of degradation, but not always enough to score a lower OHI. This indicates that OHI stages should be revisited and improved for assessing early degradation. The onset of degradation was more often observed in human ribs (17/17) than femurs (14/17). For the pigs, ribs scored a lower OHI than femurs, but the pig-only sample size was too small to apply statistics. In archaeology, some studies suggest that ribs are more sensitive predictors of inter-site variation in histological preservation [16, 73], while other studies show similar preservation between ribs and femora [45]. Unfortunately, many histotaphonomy studies target the femur without clear arguments of why this bone was chosen. Booth and Madgwick [32] and Booth [33] claim that ribs are more susceptible to biodegradation because of their location closer to the gut. While this claim should be studied by microbiologists, one could argue that the 3^rd^ rib is further away or about the same distance from the gut than the proximal end of the femur which makes this claim unstable. It is likely that time and cortical thickness have an effect on biodegradation of the bone. Hence our experimental study should be repeated over longer timeframes.

A noticeable difference between ribs and femurs was the presence of microcracks. Light microscopy did not reveal cracking, while SEM revealed more microfissures in rib sections than femoral sections (Figs 2, 3 and S1 Table). This observation argues against crack-index scoring with light microscopy only [e.g. 15] and stresses the importance of using complementary methods such as SEM. There are two types of microcracks that should be distinguished: cracks that are directly related to diagenesis and cracks that are the result of bone processing. According to Hollund et al. [21] larger cracks might have a non-diagenetic cause. In this study, both rib and femur samples were processed the same. It can be said that a rib is more fragile due to the lower ratio of cortical bone compared to femurs, but this does not explain if the higher number of microcracks is caused by sample processing or diagenesis in the field. However, the human rib sections with shorter time-since-deposition seem to have less microcracks (Fig 3C and S1 Table) which could be indicative of a taphonomic cause such as drying and sun exposure. Overall, the pig samples also showed less microcracks. It is assumed that human skeletal remains which are exposed for longer timeframes, will become dryer and more exposed to the elements. Pig bones naturally have a higher fat content protecting them longer against cracking. These observations indicate that microfissures, their shape, size, exact location and correlation with time-since-deposition should be studied in more detail to increase understanding of their cause. It is timely to develop a reproducible, automated scoring method that combines several techniques and resolutions.

A strong statistically significant correlation was noted between the MFD scores of the pigs and the humans. In addition, the pig sections showed the onset of demineralisation with SEM, which was not observed on the human sections (Fig 9C and S1 Table). While pigs are commonly used as analogues for humans when tackling other biological questions, this result stresses that pigs (and other mammals, such as rats–see [48]) cannot be used as substitute for human remains for studies of bone biodegradation because of variation in mammalian bone histology which has consequences for how biodegradation spreads throughout the bone microstructural system [48, 74]. The lateral decomposition and condensed body structure with short legs in pigs is an incorrect representation of human decomposition. Forensic comparison studies have shown that pigs have different decomposition patterns [61, 75–79], microbiome [80] and chemical markers [81] than humans, hence producing different volatile organic compounds [75] which leads to different insect activity [61].

Most MFD in bone is caused by bacteria. This goes back to the discussion about the origin of bacteria in bone; gut *versus* soil. Emmons et al. [82, 83] found that microbial communities in bone from surface-deposited and shallow buried donors were more similar to those from soils, while bones recovered from donors at the saturated, deeper areas of the grave showed increased similarity with microbial communities from human gut samples with a higher representation of anaerobic taxa. If surface depositions are more likely to be colonised by soil bacteria, this could explain the different results obtained in this study between pig and human bones and the observations that MFD was present at the side of the pig rib that was touching the soil (Fig 9). The lateral decomposition of pigs provides a direct link between soil, ribs and internal organs, while humans placed in a supine position have their spine acting as barrier between the internal organs and soil, and ribs sticking up rather than touching the ground. On the other hand, the only prone donor, also displayed an immaculate microscopic bone preservation, so this should be studied further. The reason why bone of butchered animals is better preserved microscopically in the archaeology record [9, 31] might be explained by the pre-treatment (cooking, boiling, baking, roasting) rather than their contact with soil [8]. Similarly, the well-preserved pig bones from two articulated pig carcasses deposited for three years in Turner-Walker et al.’s study [30] could be explained by the application of hydrated lime to one of the pigs [84, 85]. In addition, the depositional context of the stone-built cavity might not have favoured bacterial activity either, compared to this study where the pigs decayed in full nature. Our study demonstrates statistically significant differences in bacterial degradation of human and pigs from the same depositional context. While more research is necessary into bacterial communities in bone, it is long established that every deposition is unique due to a combination of intrinsic and extrinsic factors [40] and that the amount of oxygen in the grave is a critical factor for decomposition by aerobe bacterial communities [86–89]. The local micro-environment can change between and within each burial. Findings in the present study therefore agree with Turner-Walker [8] and Booth et al. [36] that the picture is more nuanced than bacterial degradation meaning that the bones were exposed to putrefaction.

Season of deposition, primary *versus* secondary deposition, were not statistically significantly related with bone histology markers of bioerosion. The latter confirms archaeological results obtained by Haddow et al. [14] studying histotaphonomy of 9000-year old Neolithic Near Eastern bones. It, again, stresses caution with interpretation about *in-situ* decomposition, and concluding primary burial based on the presence or absence of MFD in bone only such as done by Martinon-Torres [42] to prove the earliest human burial in Africa.

OHI and CB were both statistically significant in fresh and frozen individuals. However, looking at the human data only, there was no variability between the fresh and frozen human data. This means that these results were directly related to the human *versus* pig results, rather than the frozen *versus* fresh. More research into the effect of freezing on bone degradation is therefore necessary.

The significant correlation between OHI and CB is not surprising, nor is the link with biomolecular preservation as studied with FTIR. The intensity of birefringence is dependent on the quantity and orientation of the collagen fibres and the presence of the bone mineral [19]. Low levels of bacterial attack and no changes in organic content would not affect birefringence [19, 63, 90].

The results also indicate that there was no correlation between the post-depositional interval (days of deposition) and histological degradation, as well as between time-since-deposition and bone molecular changes. It is important to mention that the time-since-deposition ranged from 463 days (1.2 years) to 1238 days (3.3 years). From a histological point of view, Yoshino et al. [26] found that the earliest bacterial degradation in bone started between 2.5 and 5 years since death by studying non-articulated experimental bones, while a study by Bell et al. [91] noticed microstructural alterations from 3 months after death in a random forensic sample of one tibia, six ribs and three teeth ranging from 3 months to 83 years of deposition. Archaeological studies using infrared spectroscopy have shown that bone collagen decreases over time and the crystal structure of bone becomes more ordered [51, 54, 57, 63, 92–94]. In addition, the extent of chemical alteration of bone will be controlled by site specific conditions [55, 63, 94]. In this study, FTIR results showed a lack of trends at both endosteal and periosteal surfaces of pig as well as human bones (Fig 10). Looking at crystallinity index, carbonate to phosphate ratio and organic bone content, there are no correlations with time-since-deposition interval. The results are in line with the study by Wang et al. [56] who did not find a correlation between PMI and FTIR results of buried and surface skull samples deposited between 76 and 552 days (about 2.5 to 18.5 months). This is in contrast with results obtained by Howes et al [55] who found decreasing organic and carbonate contents and increasing CI of buried pig bone over a period between 3 and 23 months. However, Howes et al. [55] used disarticulated pig bones while our study examined articulated pig and human bones that underwent all stages of decomposition, which is a more realistic scenario. Trueman et al. [94] suggest that the exposure of crystal surfaces follows the loss of collagen matrix. As collagen, located in the bone matrix, degrades, the surfaces of the bone crystallites are exposed leading to an increased crystallinity. This mechanism is likely consistent with the results of the present study, and the rather short time interval of the experiment. It is assumed that the decomposition process created a protective layer of lipids, shielding collagen from degradation and bone minerals from exposure. Changes in mineral composition would only happen over a longer time interval as shown by several studies on archaeological bone samples which link reorganisation of the bone mineral’s component to time and the depositional environment [e.g. 51, 54, 92–95]. It is therefore important to conduct future studies on articulated human remains in a controlled setting over a longer skeletal time scale.

## Conclusion

This is the first, controlled, larger scale study with human remains to validate hypotheses in histotaphonomy. We demonstrate that bioerosion in bone microstructure and biochemical properties is not due to putrefaction. The results contrast with claims in the literature that bone bioerosion assessed from histology can be used to prove *in-situ* decomposition and primary burial. In this study, human donors underwent all stages of decay, including putrefaction. The bones were well-preserved on a microscopic level and did not show changes in crystallinity and organic content which makes PMI prediction unreliable over the studied period of time. These results have a significant implication for the interpretation of early post-mortem practices in archaeology and forensic science.

A second important outcome is that pig remains cannot be used as proxy of human remains for the study of bone histotaphonomy. Variation was noted between human and pig histology preservation despite originating from the same experimental context.

Finally, this study demonstrates that optical microscopy of thin sections remains a principal technique for the evaluation of diagenetic changes, but to understand the complete suite of biological, physical and chemical changes, electron microscopy and IR spectroscopy should also be used.

The main flaws in former studies on histotaphonomy are that they used unvalidated assumptions and confused several parameters, such as *in-situ* decay, putrefaction and bacterial origin, in their study design and interpretations. Additional factors that complicate archaeological studies of histotaphonomy are the unknown depositional timelines and contextual uncertainties. The key message from the present study is a reminder that taphonomy is multi-factorial and approaches such as histotaphonomy require rigorous experimental validation before they can be used to make conclusions about ancient patterns in funerary treatment. It is not soil *versus* gut bacteria, neither is bioerosion *only* related to early post-mortem events. These results highlight that human remains should be analysed in their broader depositional context on a case-by-case basis, and emphasises a self-critical use of methods and interpretations. This study is currently being expanded to a similar larger scale study at different locations (Canada) and different settings (buried vs surface) over a longer period of time.

## Supporting information

S1 Table In situ images of each subject (humans and pigs) on the day of sampling, with entire thin sections auto-stitched at x100 magnification in transmitted and polarising light, and additional SEM images of each sample taken at x350 magnification. (PDF)

S2 Table Overview of all OHI and CB scores, assessed by the two observers (ES, JM). (PDF)

S1 File Statistical details. (PDF)
