# Supplementary material for: Unravelling taphono-myths. First large-scale study of histotaphonomic changes and diagenesis in bone from modern surface depositions
Source: PLoS One. 2024 Sep 26;19(9):e0308440. doi: 10.1371/journal.pone.0308440 (PMC11426454; doi:10.1371/journal.pone.0308440)
Supplement: S1 Table — (PDF) [file pone.0308440.s001.pdf]

Donor No.	Sample No.	Rib (R) Femur (F)	Subject in its depositional environment (photo taken on sample day)	Thin section under transmitted light at x100 magnification	Thin section under polarised light at x100 magnification	SEM image at x350 magnification
1602	HB03	R	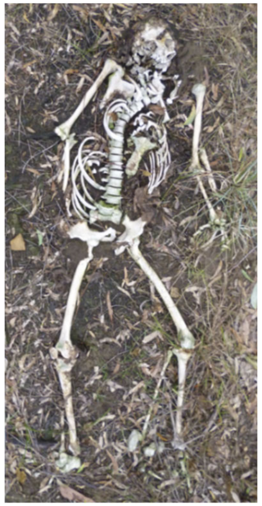	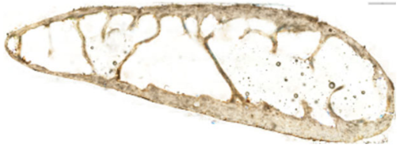	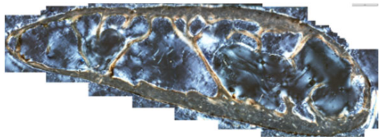	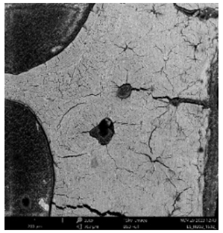
	HB04	F		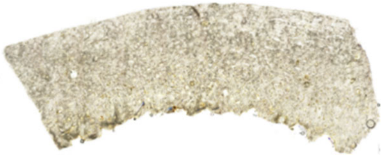	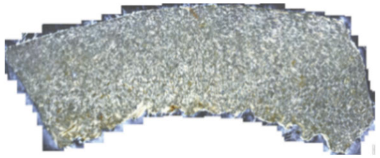	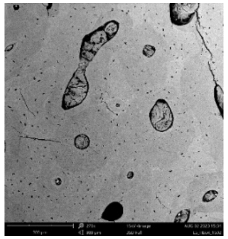
1603	HB05	F	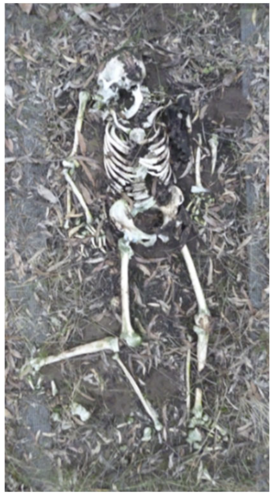	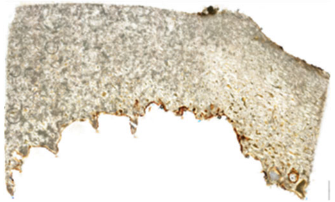	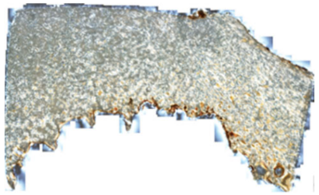	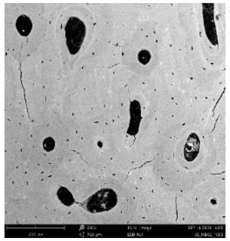
	HB06	R		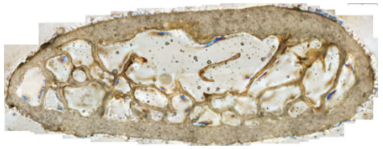	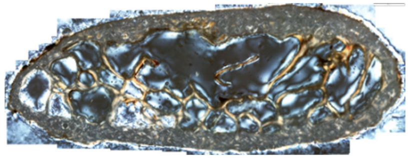	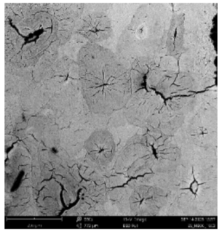

1617	HB07	F	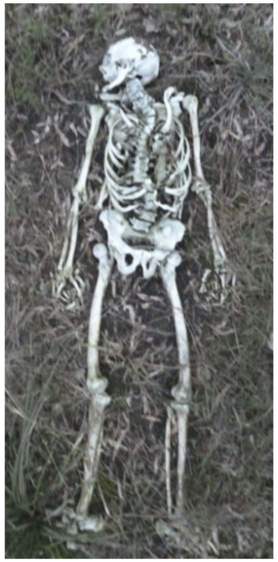	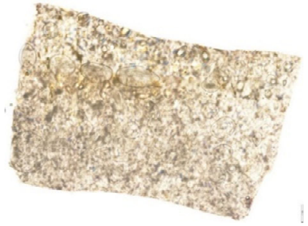	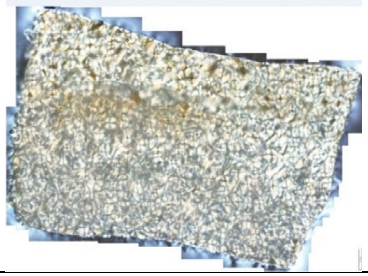	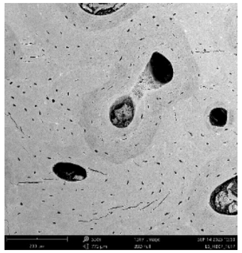
	HB08	R		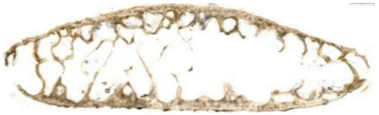	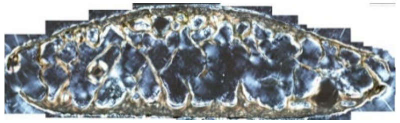	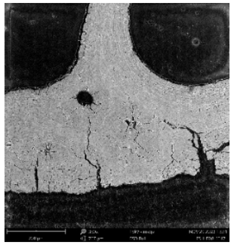
1618	HB09	F	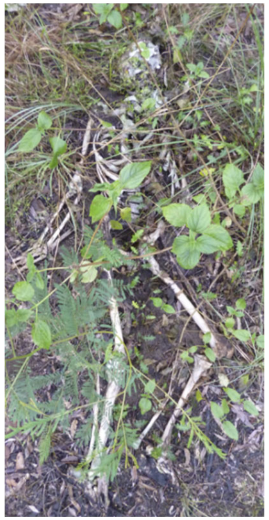	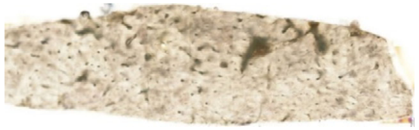	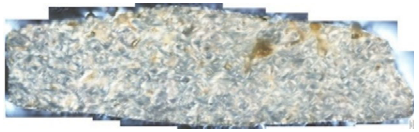	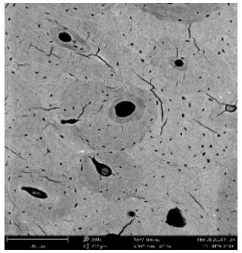
	HB10	R		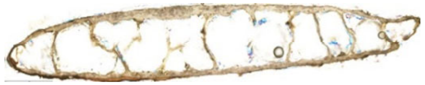	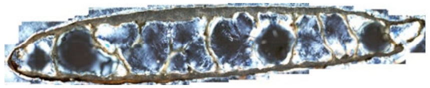	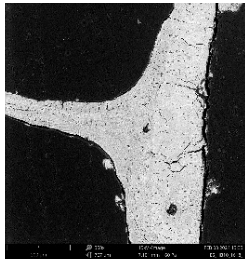

1619	HB11	F	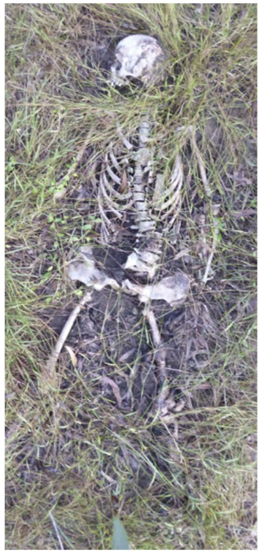	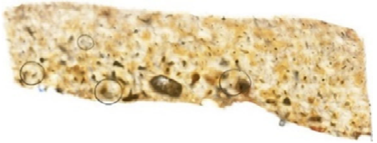	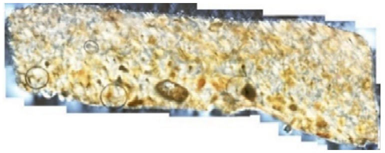	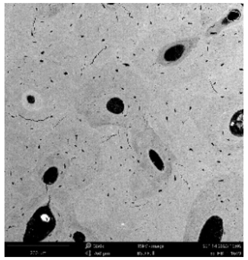
	HB12	R		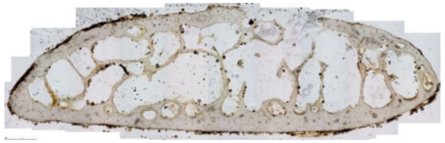	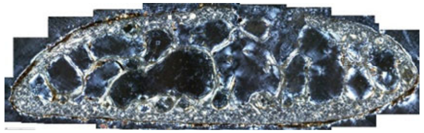	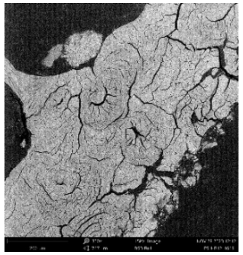
1705	HB13	F	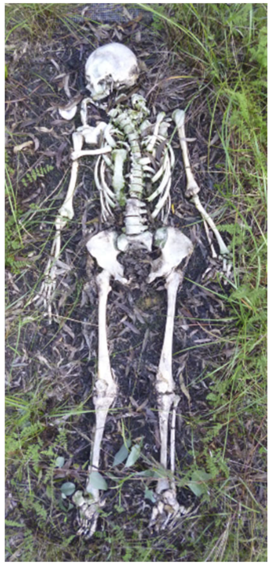	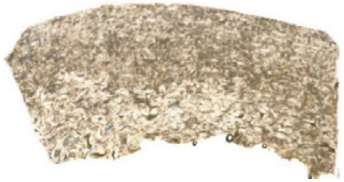	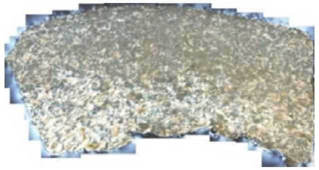	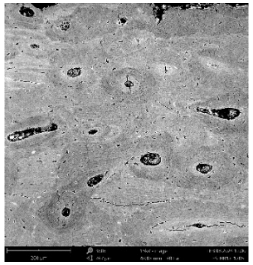
	HB14	R		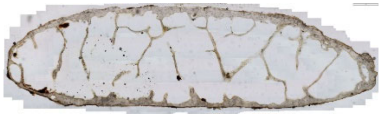	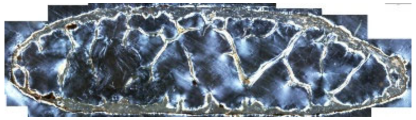	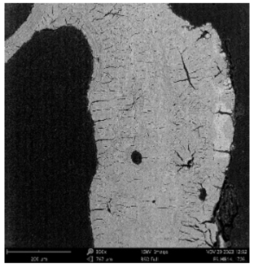

1621	HB15	F	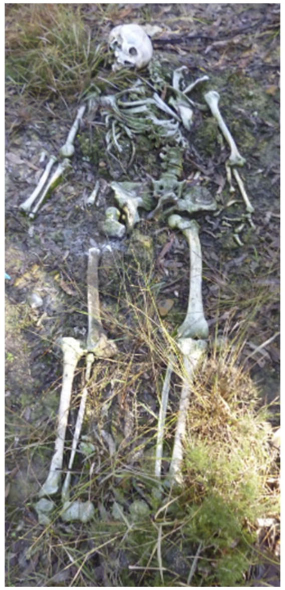	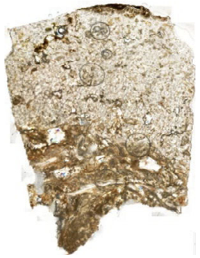	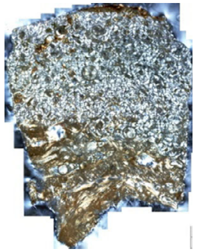	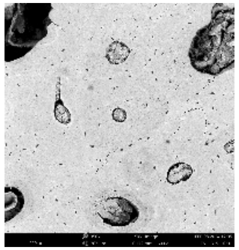
	HB16	R		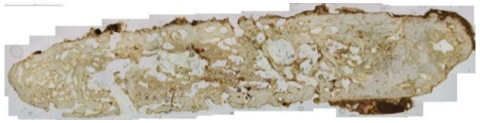	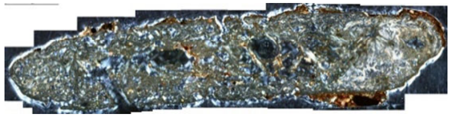	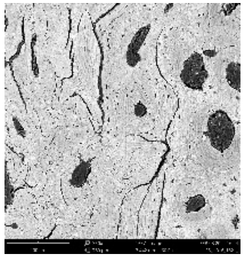
1716	HB17	F	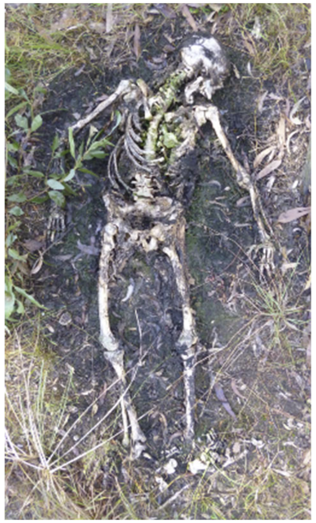	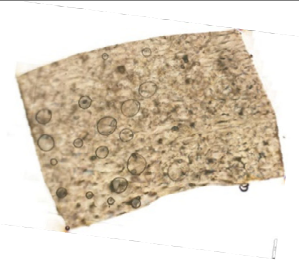	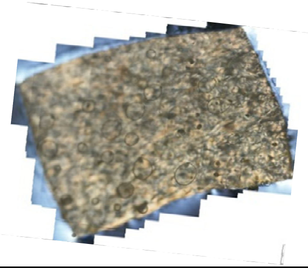	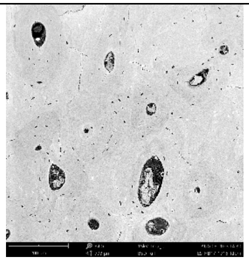
	HB18	R		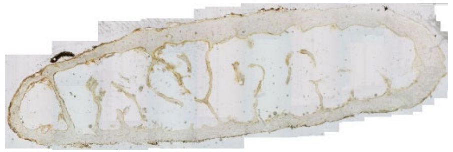	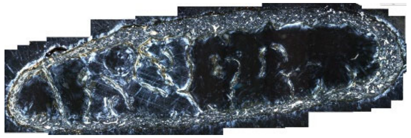	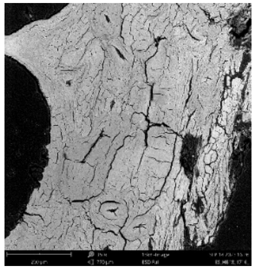

1722	HB19	F	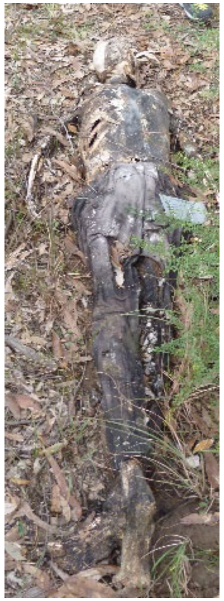	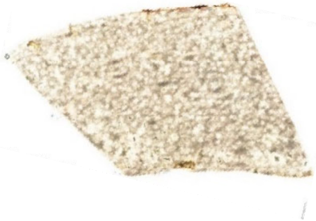	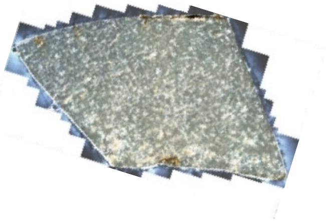	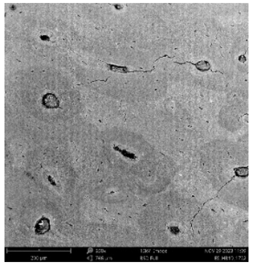
	HB20	R		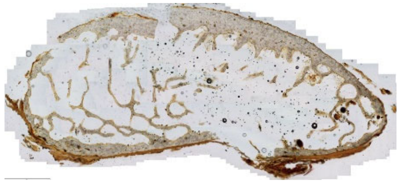	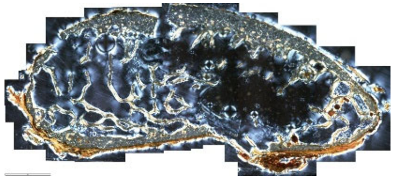	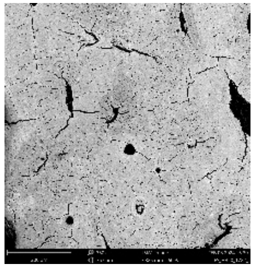
1718	HB21	R	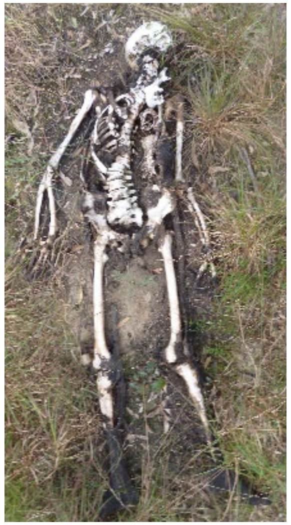	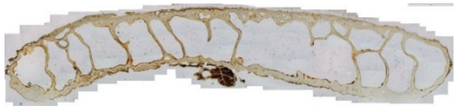	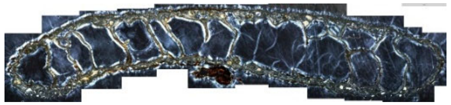	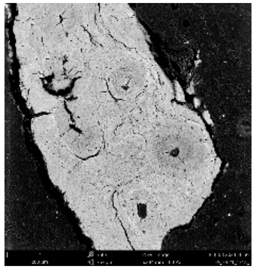
	HB22	F		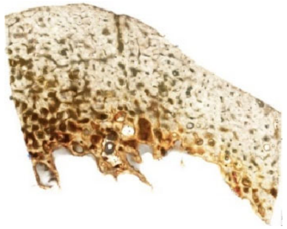	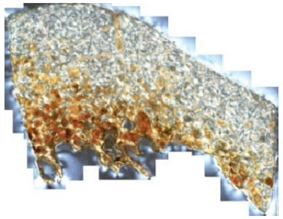	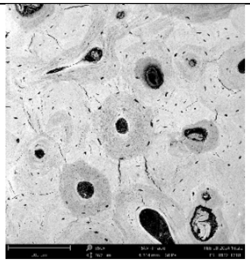

1717	HB23	F	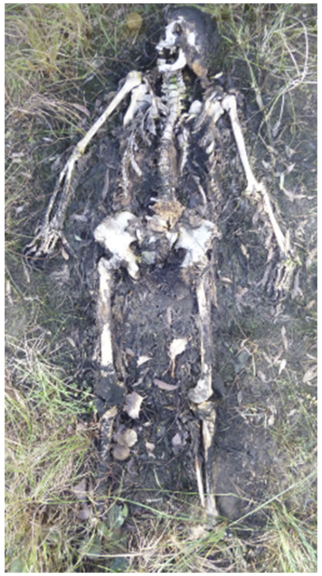	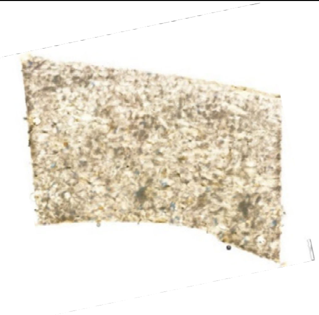	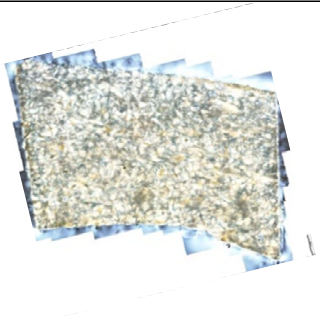	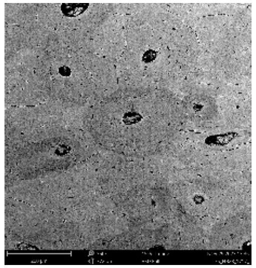
	HB24	R		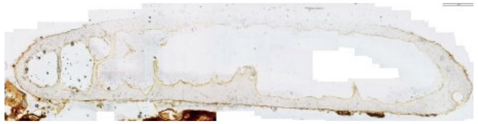	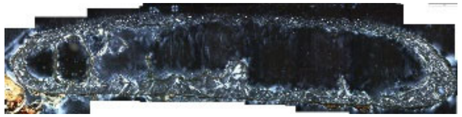	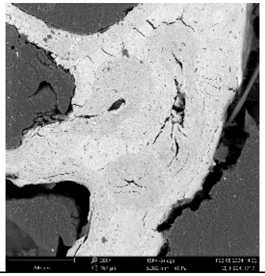
1721	HB25	F	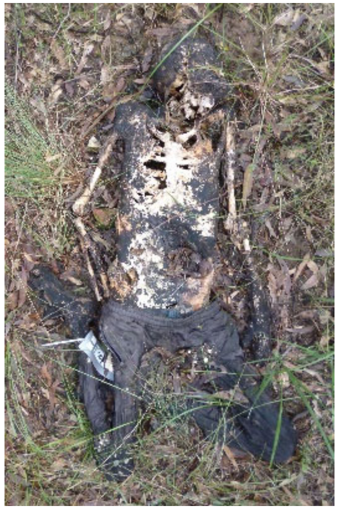	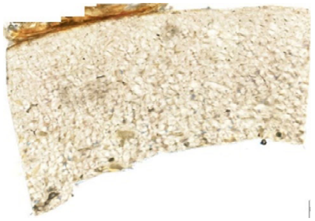	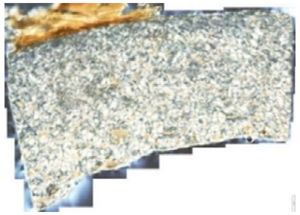	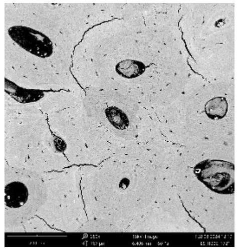
	HB26	R		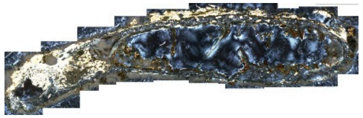	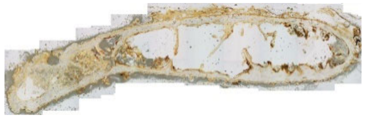	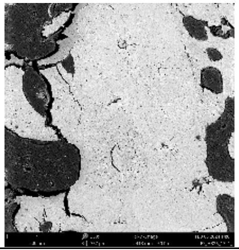

1805	HB27	F	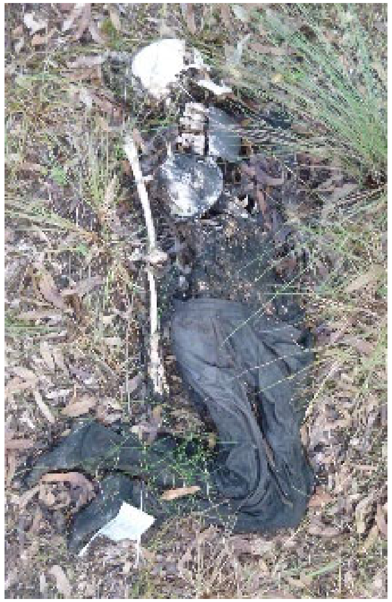	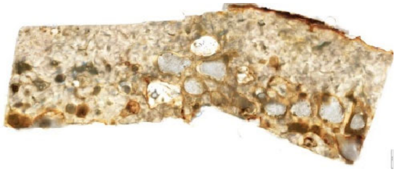	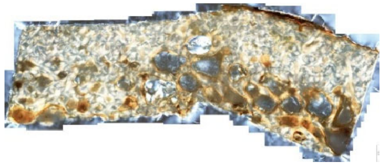	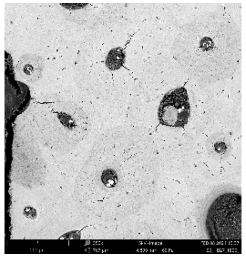
	HB28	R		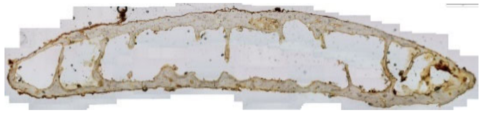	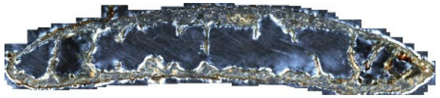	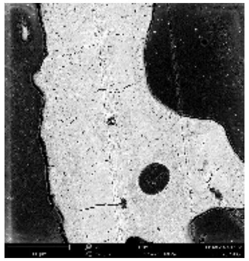
1806	HB29	F	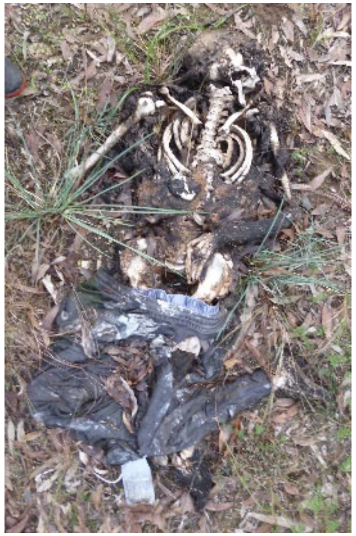	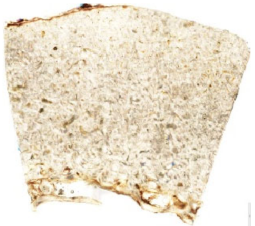	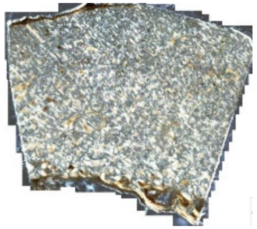	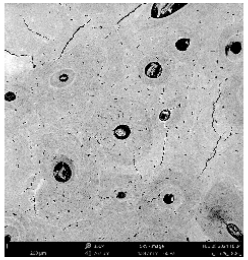
	HB30	R		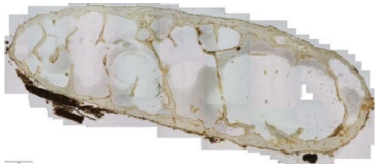	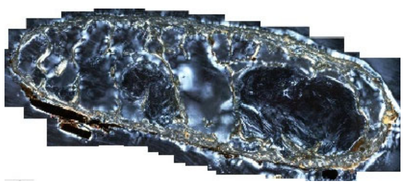	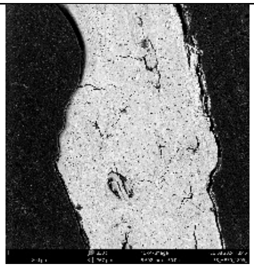

1706	HB33	F	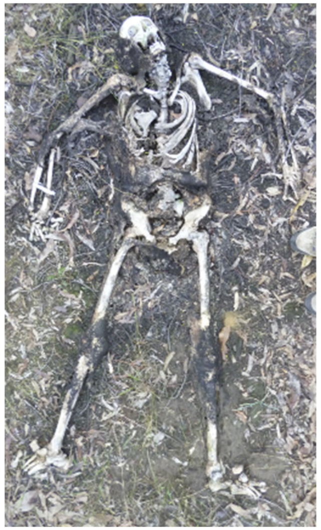	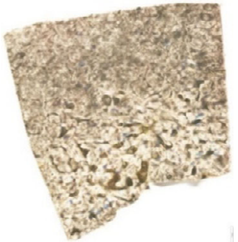	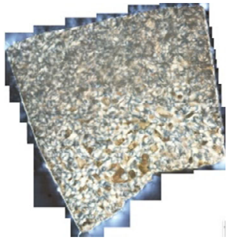	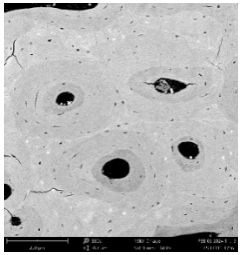
	HB34	R		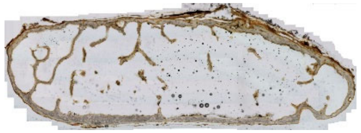	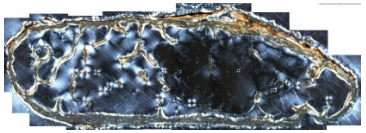	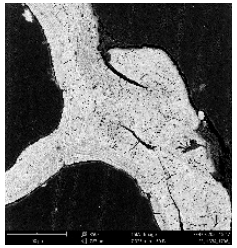
1719	HB35	F	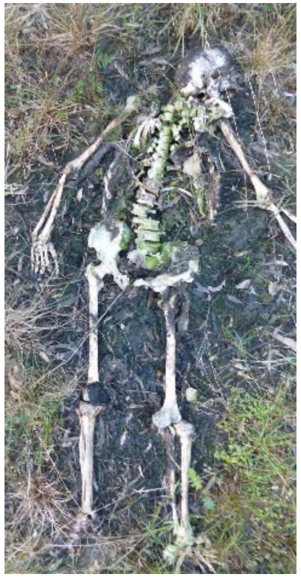	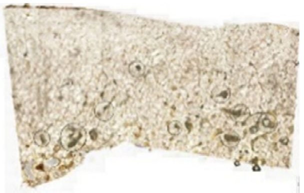	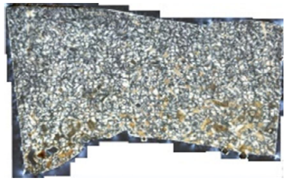	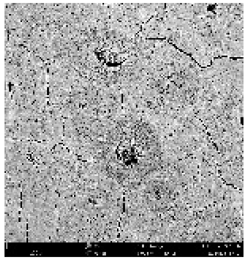
	HB36	R		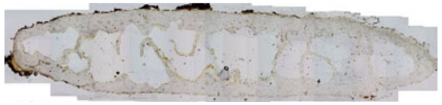	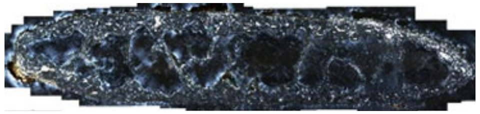	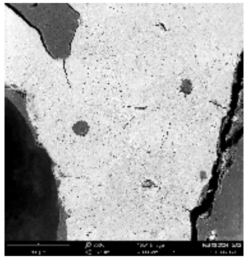

1803	HB37	F	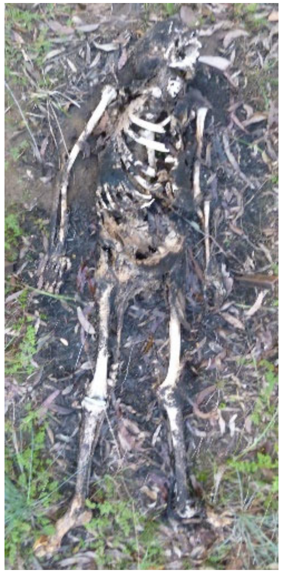	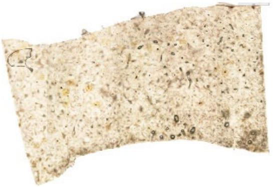	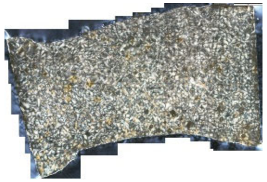	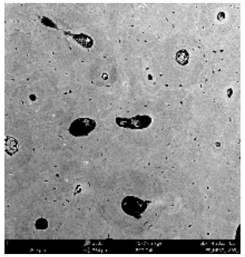
	HB38	R		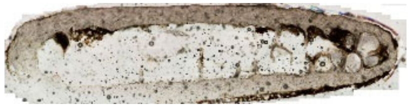	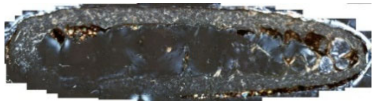	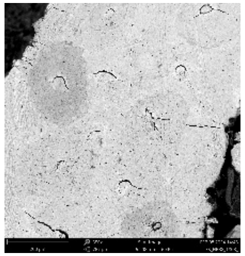
4041	PB40	F	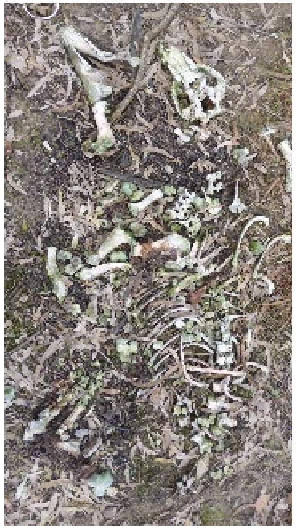	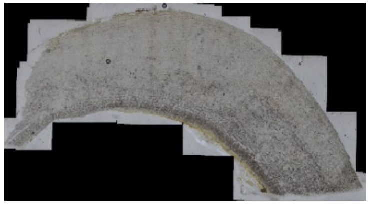	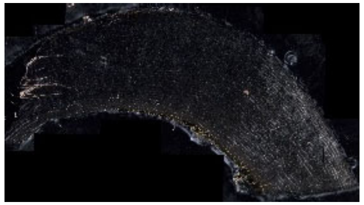	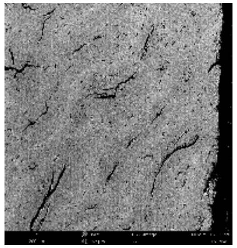
	PB41	R		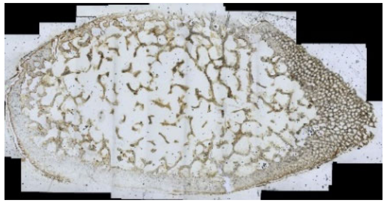	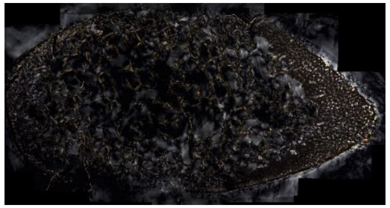	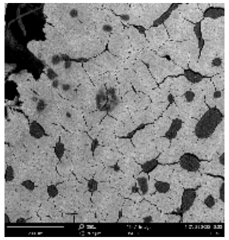

4243	PB42	F	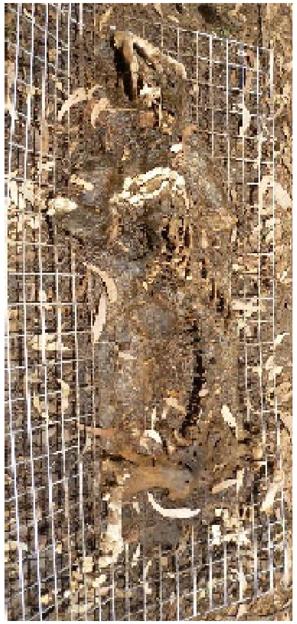	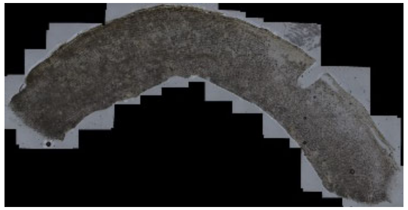	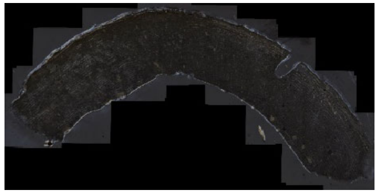	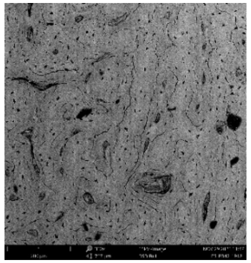
	PB43	R		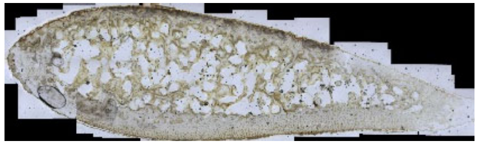	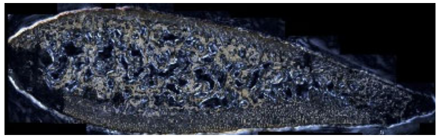	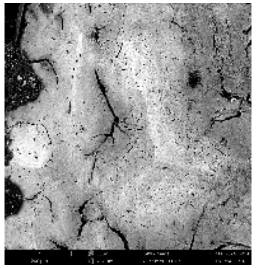
4446	PB44	F	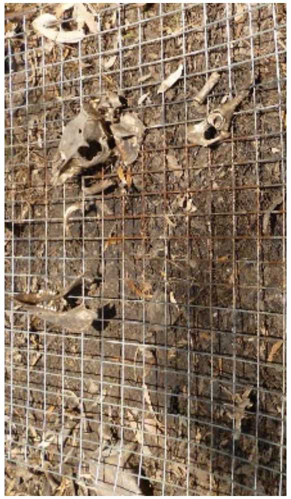	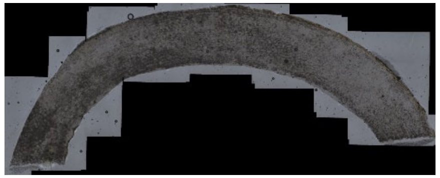	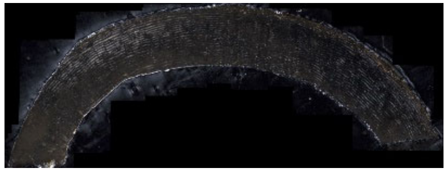	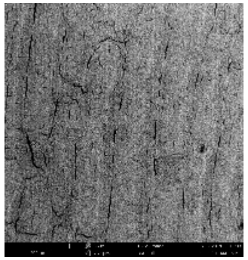
	PB46	R		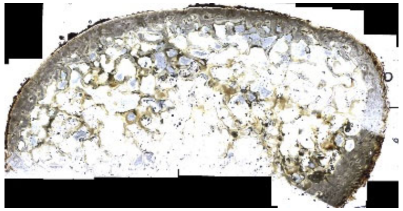	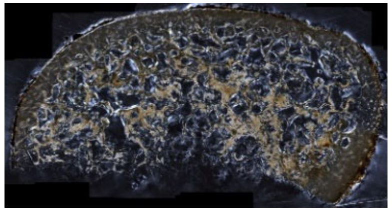	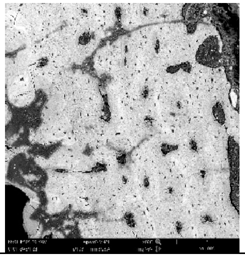

4849	PB48	F	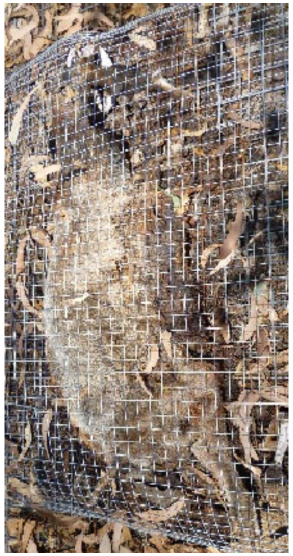	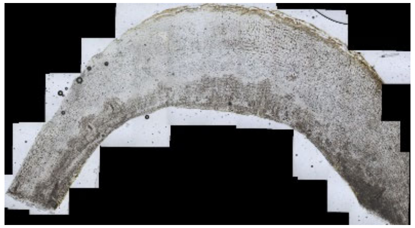	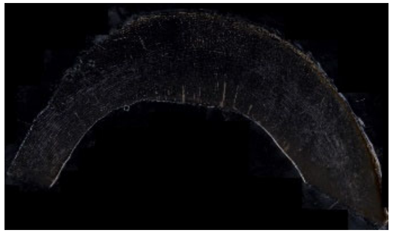	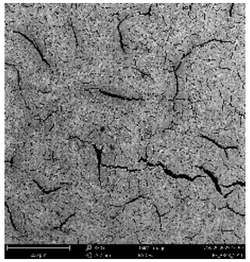
	PB49	R		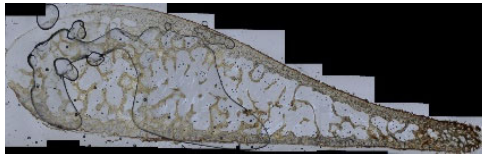	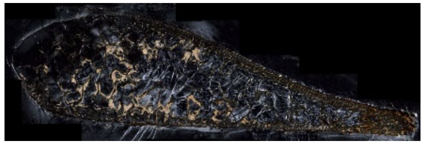	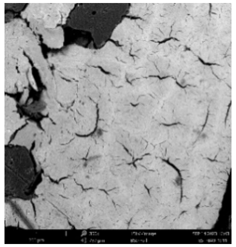
5051	PB50	F	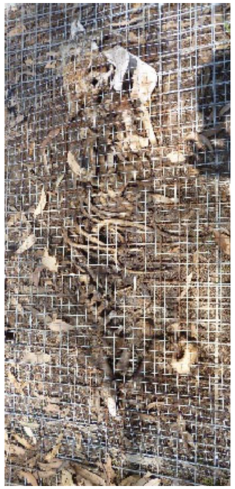	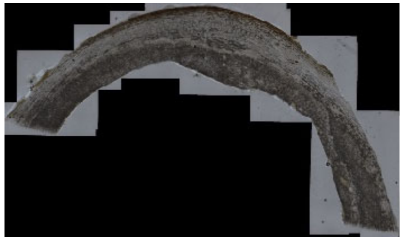	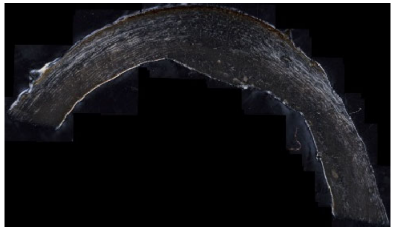	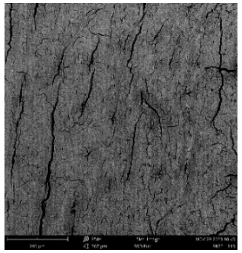
	PB51	R		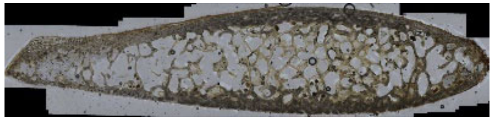	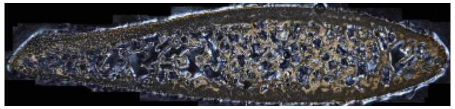	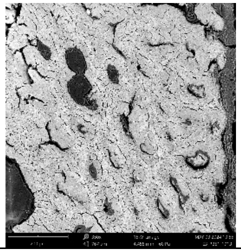

3902	PB02	F	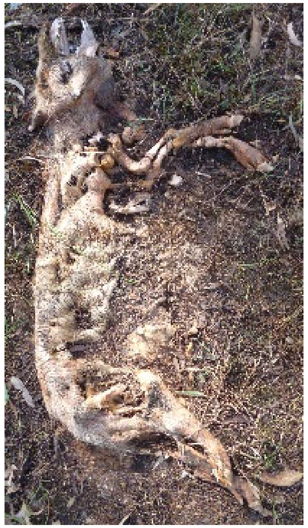	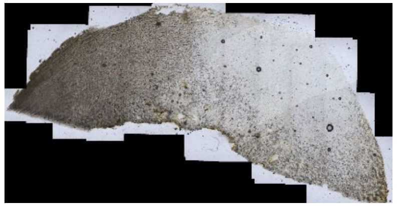	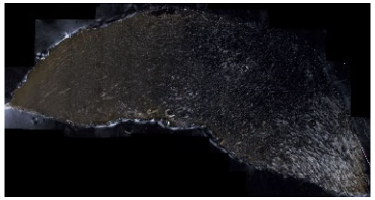	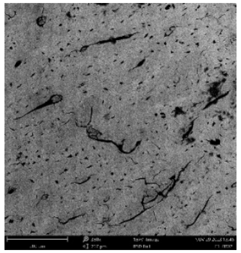
	PB39	R		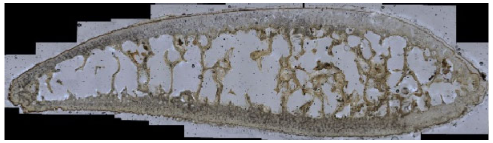	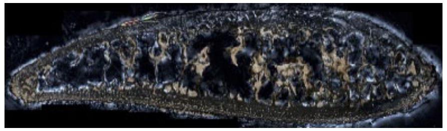	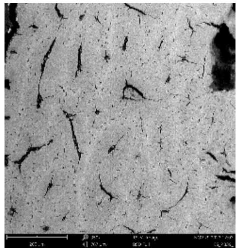
