# Supplementary material for: Unravelling taphono-myths. First large-scale study of histotaphonomic changes and diagenesis in bone from modern surface depositions
Source: PLoS One. 2024 Sep 26;19(9):e0308440. doi: 10.1371/journal.pone.0308440 (PMC11426454; doi:10.1371/journal.pone.0308440)
Supplement: S2 Table — (PDF) [file pone.0308440.s002.pdf]

			JM1		JM2		JM3		ES1		ES2		ES3	
	Sample number (2 samples per donor)	Donor number	OHI (0 to 5)	CB (0 - 0.5 - 1)	OHI (0 to 5)	CB (0 - 0.5 - 1)	OHI (0 to 5)	CB (0 - 0.5 - 1)	OHI (0 to 5)	CB (0 - 0.5 - 1)	OHI (0 to 5)	CB (0 - 0.5 - 1)	OHI (0 to 5)	CB (0 - 0.5 - 1)
P1	PB02-3902	3902	3	0.5	3	0.5	3	0.5	3	0.5	3	0.5	3	0.5
P2	PB39-3902	3902	3	0.5	3	0.5	3	0.5	4	0.5	3	0.5	4	0.5
1	HB03-1602	1602	5	1	5	1	5	1	5	1	5	0.5	5	0.5
2	HB04-1602	1602	5	1	5	1	5	1	5	1	5	1	5	1
3	HB05-1603	1603	5	1	5	1	5	1	5	1	5	1	5	1
4	HB06-1603	1603	5	1	5	1	5	1	5	1	5	0.5	5	0.5
5	HB07-1617	1617	5	1	5	1	5	1	5	1	5	1	5	1
6	HB08-1617	1617	5	1	5	1	5	1	5	1	5	1	5	1
7	HB09-1618	1618	5	1	5	1	5	1	5	1	5	1	5	1
8	HB10-1618	1618	5	1	5	1	5	1	5	1	5	0.5	5	0.5
9	HB11-1619	1619	5	1	5	1	5	1	5	1	5	1	5	1
10	HB12-1619	1619	5	1	5	1	5	1	5	1	5	1	4	1
11	HB13-1705	1705	5	1	5	1	5	1	5	1	5	1	5	1
12	HB14-1705	1705	5	1	5	1	5	1	5	1	5	0.5	4	1
13	HB15-1621	1621	5	1	5	1	5	1	5	1	5	1	5	1
14	HB16-1621	1621	5	1	5	1	5	1	5	1	5	1	4	1
15	HB17-1716	1716	5	1	5	1	5	1	5	1	5	1	5	1
16	HB18-1716	1716	5	1	5	1	5	1	5	1	5	1	5	1
17	HB19-1722	1722	5	1	5	1	5	1	5	1	5	1	5	1
18	HB20-1722	1722	5	1	5	1	5	1	5	1	5	1	5	1
19	HB21-1718	1718	5	1	5	1	5	1	5	1	5	1	5	1
20	HB22-1718	1718	5	1	5	1	5	1	5	1	5	1	5	1
21	HB23-1717	1717	5	1	5	1	5	1	5	1	5	1	5	1
22	HB24-1717	1717	5	1	5	1	5	1	5	1	5	1	5	1
23	HB25-1721	1721	5	1	5	1	5	1	5	1	5	1	5	1
24	HB26-1721	1721	5	1	5	1	5	1	5	1	5	1	5	1
25	HB27-1805	1805	5	1	5	1	5	1	5	1	5	1	5	1
26	HB28-1805	1805	5	1	5	1	5	1	5	1	5	1	5	1
27	HB29-1806	1806	5	1	5	1	5	1	5	1	5	1	5	1
28	HB30-1806	1806	5	1	5	1	5	1	5	1	5	1	5	1
29	HB33-1706	1706	5	1	5	1	5	1	5	1	5	1	5	1
30	HB34-1706	1706	5	1	5	1	5	1	5	1	5	1	5	1
31	HB35-1719	1719	5	1	5	1	5	1	5	1	5	1	5	1
32	HB36-1719	1719	5	1	5	1	5	1	5	1	5	1	5	1
33	HB37-1803	1803	5	1	5	1	5	1	5	1	5	1	5	1
34	HB38-1803	1803	5	1	5	1	5	1	5	1	5	1	4	0.5
P3	PB40-4041	4041	3	1	3	1	4	1	4	1	4	1	4	0.5
P4	PB41-4041	4041	3	0.5	3	0.5	3	0.5	3	0.5	3	0.5	3	0.5
P5	PB42-1918	4243	3	0	3	0	3	0	2	0	2	0	2	0
P6	PB43-1918	4243	4	0.5	4	0.5	4	0.5	4	0.5	4	0.5	4	0.5
P7	PB44-1919	4446	3	0.5	3	0.5	3	0.5	3	0.5	3	0.5	3	0.5
P8	PB46-1919	4446	2	0.5	2	0.5	2	0.5	2	0.5	2	0.5	2	1
P9	PB48-1911	4849	4	0	4	0	4	0	4	0	4	0.5	4	0.5
P10	PB49-1911	4849	4	0.5	4	0.5	4	0.5	4	0.5	4	0.5	3	0.5
P11	PB50-1913	5051	3	0.5	3	0.5	3	0.5	3	0.5	3	0.5	3	0.5
P12	PB51-1913	5051	2	0.5	2	0.5	2	0.5	2	0.5	2	0.5	2	0.5
