# Supplementary material for: Unravelling taphono-myths. First large-scale study of histotaphonomic changes and diagenesis in bone from modern surface depositions
Source: PLoS One. 2024 Sep 26;19(9):e0308440. doi: 10.1371/journal.pone.0308440 (PMC11426454; doi:10.1371/journal.pone.0308440)
Supplement: S1 File — (PDF) [file pone.0308440.s003.pdf]

Supplementary S3

Statistical tests

OHI Analysis

Pig or Human

Pig or Human	bin_OHI 5	<5	Total
Human	34	0	34
	100.00	0.00	73.91
Pig	0	12	12
	0.00	100.00	26.09
Total	34	12	46
	100.00	100.00	100.00

Fisher's exact = 0.000

Femur or Rib

Femur or Rib	bin_OHI 5	<5	Total
Femur	17	6	23
	50.00	50.00	50.00
Rib	17	6	23
	50.00	50.00	50.00
Total	34	12	46
	100.00	100.00	100.00

Fisher's exact = 1.000

Frozen or Fresh

Frozen	bin_OHI 5	<5	Total
No	22	12	34
	64.71	100.00	73.91
Yes	12	0	12
	35.29	0.00	26.09
Total	34	12	46
	100.00	100.00	100.00

Fisher's exact = 0.020

Season of deposition

Season of deposition	bin_OHI 5	<5	Total
Autumn	12	4	16
	35.29	33.33	34.78
Spring	2	4	6
	5.88	33.33	13.04
Summer	6	2	8
	17.65	16.67	17.39
Winter	14	2	16
	41.18	16.67	34.78
Total	34	12	46
	100.00	100.00	100.00

Fisher's exact = 0.107

Secondary Placement

Secondary placement	bin_OHI 5	bin_OHI <5	Total
No	26	12	38
	76.47	100.00	82.61
Yes	8	0	8
	23.53	0.00	17.39
Total	34	12	46
	100.00	100.00	100.00

Fisher's exact = 0.090

Days of deposition

Logistic regression — Number of obs = 46

Wald chi2(1) = 3.53

Prob > chi2 = 0.0800

Log pseudolikelihood = -10.173904 — Pseudo R2 = 0.6147

(Std. err. adjusted for 23 clusters in id)

bin_OHI	Odds ratio	Robust std. err.	z	P>|z|	[95% conf.	interval]
DaysofdepositionPMI	.9863225	.0072319	-1.88	0.080	.9722496	1.000599
_cons	292.8973	821.0334	2.03	0.043	1.204133	71245.31

CB Analysis

Pig or Human

Pig or Human	bin_CB 1	<1	Total
H	34	0	34
	97.14	0.00	73.91
P	1	11	12
	2.86	100.00	26.09
Total	35	11	46
	100.00	100.00	100.00

Fisher's exact = 0.000

Femur or Rib

Femur or Rib	bin_CB 1	<1	Total
Femur	18	5	23
	51.43	45.45	50.00
Rib	17	6	23
	48.57	54.55	50.00
Total	35	11	46
	100.00	100.00	100.00

Fisher's exact = 1.000

Frozen or Fresh

Frozen	bin_CB 1	<1	Total
No	23	11	34
	65.71	100.00	73.91
Yes	12	0	12
	34.29	0.00	26.09
Total	35	11	46
	100.00	100.00	100.00

Fisher's exact = 0.044

Season of deposition

Season of deposition	bin_CB 1	<1	Total
Autumn	12	4	16
	34.29	36.36	34.78
Spring	2	4	6
	5.71	36.36	13.04
Summer	7	1	8
	20.00	9.09	17.39
Winter	14	2	16
	40.00	18.18	34.78
Total	35	11	46
	100.00	100.00	100.00

Fisher's exact = 0.075

Secondary Placement

Secondary placement	bin_CB 1	<1	Total
No	27	11	38
	77.14	100.00	82.61
Yes	8	0	8
	22.86	0.00	17.39
Total	35	11	46
	100.00	100.00	100.00

Fisher's exact = 0.169

Days of deposition

```
Logistic regression                                    Number of obs =     46
                                                       Wald chi2(1)  =   2.45
                                                       Prob > chi2   = 0.1173
Log pseudolikelihood = -7.2734254                      Pseudo R2     = 0.7126

                                    (Std. err. adjusted for 23 clusters in id)
------------------------------------------------------------------------------
                    |               Robust
             bin_CB | Odds ratio   std. err.      z    P>|z|     [95% conf. interval]
--------------------+---------------------------------------------------------
DaysofdepositionPMI |   .9796875    .012835    -1.57   0.117     .9548516    1.005169
              _cons |   3994.517   21729.69     1.52   0.127     .0935471    1.71e+08
```

Inter Rater Agreement

Obs1	Obs2: 2	3	4	5	Total
2	6	0	0	0	6
3	3	13	4	0	20
4	0	1	9	0	10
5	0	0	4	98	102
Total	9	14	17	98	138

Interrater agreement

Number of ratings = 138
Number of raters = 2
Number of rating categories = 4

	Coef.	Std. Err.	t	P>|t|	[95% Conf.	Interval]
Percent Agreement	0.9130	0.0241	37.93	0.000	0.8654	0.9606
Cohen/Conger's Kappa	0.8062	0.0491	16.43	0.000	0.6908	0.9216

Benchmark scale

<0.0000	Poor
0.0000-0.2000	Slight
0.2000-0.4000	Fair
0.4000-0.6000	Moderate
0.6000-0.8000	Substantial
0.8000-1.0000	Almost Perfect

Intra rater Agreement

-> observer = 1

Intraclass correlations
Two-way random-effects model - Absolute agreement

Random effects: id   Number of targets = 23
Fixed effects: time   Number of ratings/target = 6

OHI_Measmt	ICC	[95% conf.	interval]
Individual	.9435776	.9020856	.9722267

F test that
ICC=0.00: F(22.0, 110.0) = 101.34   Prob > F = 0.000

-> observer = 2

```
Intraclass correlations
Two-way random-effects model - Absolute agreement

Random effects: id              Number of targets =         23
Fixed effects: time       Number of ratings/target =          6

--------------------------------------------------------------
            OHI_Measmt |        ICC       [95% conf. interval]
-----------------------+--------------------------------------
            Individual |   .8428894       .7439328    .9190447
--------------------------------------------------------------
F test that
  ICC=0.00: F(22.0, 110.0) = 33.19            Prob > F = 0.000
```

Biomolecular results

Crystallinity index (CI)

Humans

sample	PMI /days	Human Inner		PMI /days	Human Outer
HB19	463	3.25		463	3.18
HB25	463	3.54		463	3.42
HB27	463	3.24		463	3.18
HB29	463	3.10		463	3.27
HB37	531	3.23		531	3.22
HB35	714	3.26		714	3.36
HB22	717	3.22		717	3.28
HB23	718	3.21		718	3.29
HB17	725	3.33		725	3.64
HB33	830	3.34		830	3.44
HB13	836	3.17		836	3.27
HB15	949	3.32		949	3.39
HB11	1057	3.23		1057	3.35
HB07	1062	3.16		1062	3.15
HB09	1063	3.13		1063	3.34
HB05	1238	3.23		1238	3.34
HB04	1238	3.16		1238	3.29

Human Inner

Regression Statistics	
Multiple R	0.314508523
R Square	0.098915611
Adjusted R Square	0.038843318
Standard Error	0.099831592
Observations	17

ANOVA

	df	SS	MS	F	Significance F
Regression	1	0.016410682	0	1.646609552	0.21889302
Residual	15	0.149495201	0		
Total	16	0.165905882			

	Coefficients	Standard Error	Sta	P-value	Lower 95%	Upper 95%	Lower 95.0%	Upper 95.0%
Intercept	3.336816068	0.07749478	43	3.90612E-17	3.171639853	3.501992282	3.171639853	3.501992282
PMI /days	-0.00011869	9.2495E-05	-1	0.21889302	-0.000315838	7.84586E-05	-0.000315838	7.84586E-05

Human Outer

Regression Statistics	
Multiple R	0.115978683
R Square	0.013451055
Adjusted R Square	-0.052318875
Standard Error	0.120683708
Observations	17

ANOVA

	df	SS	MS	F	Significance F
Regression	1	0.002978697	0	0.204516791	0.6575707
Residual	15	0.218468362	0		
Total	16	0.221447059			

	Coefficients	Standard Error	Sta	P-value	Lower 95%	Upper 95%	Lower 95.0%	Upper 95.0%
Intercept	3.277990275	0.093681342	35	8.52505E-16	3.078313222	3.477667329	3.078313222	3.477667329
PMI /days	5.05665E-05	0.000111815	0	0.6575707	-0.000187761	0.000288894	-0.000187761	0.000288894

Pigs

sample	PMI /da	Pig Inner		PMI /da	Pig Out
PB44	78	3.31		78	3.16
PB42	83	3.44		83	3.33
PB50	237	3.35		237	3.23
PB48	268	3.58		268	4.65
PB40	583	3.08		583	3.41

Pig Inner

Regression Statistics	
Multiple R	0.620947004
R Square	0.385575182
Adjusted R Square	0.18076691
Standard Error	0.166575583
Observations	5

ANOVA

	df	*SS*	*MS*	*F*	*Significance F*
Regression	1	0.052237726	0.052237726	1.882615274	0.263633904
Residual	3	0.083242274	0.027747425		
Total	4	0.13548			

	Coefficients	*Standard Error*	*t Stat*	*P-value*	*Lower 95%*	*Upper 95%*	*Lower 95.0%*	*Upper 95.0%*
Intercept	3.490940181	0.12571199	27.76934941	0.000102506	3.090868522	3.891011839	3.090868522	3.891011839
PMI /days	-0.000556206	0.000405373	-1.372084281	0.263633904	-0.001846283	0.000733872	-0.001846283	0.000733872

Pig Outer

Regression Statistics	
Multiple R	0.159566862
R Square	0.025461583
Adjusted R Square	-0.299384556
Standard Error	0.70552692
Observations	5

ANOVA

	df	*SS*	*MS*	*F*	*Significance F*
Regression	1	0.039015293	0.039015293	0.07838044	0.797698643
Residual	3	1.493304707	0.497768236		
Total	4	1.53232			

	Coefficients	*Standard Error*	*t Stat*	*P-value*	*Lower 95%*	*Upper 95%*	*Lower 95.0%*	*Upper 95.0%*
Intercept	3.435924842	0.532450146	6.453045172	0.007548311	1.741430843	5.130418841	1.741430843	5.130418841
PMI /days	0.000480685	0.001716947	0.279965069	0.797698643	-0.004983406	0.005944777	-0.004983406	0.005944777

Carbonate to phosphate ratio (C/P)

Humans

sample	PMI /days	Human Inner		PMI /days	Human Outer
HB19	463	0.53		463	0.47
HB25	463	1.06		463	1.65
HB27	463	0.60		463	0.51
HB29	463	0.73		463	0.45
HB37	531	0.52		531	0.50
HB35	714	0.62		714	0.39
HB22	717	0.65		717	0.44
HB23	718	0.53		718	0.50
HB17	725	0.51		725	1.09
HB33	830	0.36		830	0.58
HB13	836	0.62		836	0.39
HB15	949	0.49		949	0.34
HB11	1057	0.60		1057	0.38
HB07	1062	0.58		1062	0.56
HB09	1063	0.59		1063	0.43
HB05	1238	0.80		1238	0.40
HB04	1238	0.61		1238	0.39

Human Inner

Regression Statistics	
Multiple R	0.130579978
R Square	0.017051131
Adjusted R Square	-0.048478794
Standard Error	0.1544538
Observations	17

ANOVA

	df	SS	MS	F	Significance F
Regression	1	0.006207414	0	0.260203729	0.617403601
Residual	15	0.357839645	0		
Total	16	0.364047059			

	Coefficients	Standard Error	Sta	P-value	Lower 95%	Upper 95%	Lower 95.0%	Upper 95.0%
Intercept	0.669861793	0.119895547	6	5.18904E-05	0.414310484	0.925413102	0.414310484	0.925413102
PMI /days	-7.29971E-05	0.000143103	-1	0.617403601	-0.000378014	0.00023202	-0.000378014	0.00023202

Human Outer

Regression Statistics	
Multiple R	0.381577576
R Square	0.145601447
Adjusted R Square	0.088641543
Standard Error	0.313326362
Observations	17

ANOVA

	df	SS	MS	F	Significance F
Regression	1	0.250951802	0	2.556209503	0.130708849
Residual	15	1.472601139	0		
Total	16	1.723552941			

	Coefficients	Standard Error	Sta	P-value	Lower 95%	Upper 95%	Lower 95.0%	Upper 95.0%
Intercept	0.926456503	0.243221181	4	0.001711061	0.408042829	1.444870178	0.408042829	1.444870178
PMI /days	-0.000464136	0.0002903	-2	0.130708849	-0.001082896	0.000154624	-0.001082896	0.000154624

Pigs

sample	PMI /days	Pig Inner		PMI /days	Pig Outer
PB44	78	0.50		78	0.45
PB42	83	0.66		83	0.49
PB50	237	0.57		237	0.50
PB48	268	0.62		268	1.00
PB40	583	0.49		583	0.40

Pig Inner								
Regression Statistics								
Multiple R	0.472699882							
R Square	0.223445178							
Adjusted R Square	-0.035406429							
Standard Error	0.075257379							
Observations	5							
ANOVA								
	df	*SS*	*MS*	*F*	*Significance F*			
Regression	1	0.00488898	0.00488898	0.863217271	0.421372283			
Residual	3	0.01699102	0.005663673					
Total	4	0.02188						
	Coefficients	*Standard Error*	*t Stat*	*P-value*	*Lower 95%*	*Upper 95%*	*Lower 95.0%*	*Upper 95.0%*
Intercept	0.610505476	0.056795569	10.74917445	0.001721778	0.429756628	0.791254323	0.429756628	0.791254323
PMI /days	-0.000170158	0.000183144	-0.929094867	0.421372283	-0.000753004	0.000412688	-0.000753004	0.000412688

Pig Outer								
Regression Statistics								
Multiple R	0.069431461							
R Square	0.004820728							
Adjusted R Square	-0.326905696							
Standard Error	0.281854296							
Observations	5							
ANOVA								
	df	*SS*	*MS*	*F*	*Significance F*			
Regression	1	0.001154468	0.001154468	0.014532239	0.911668197			
Residual	3	0.238325532	0.079441844					
Total	4	0.23948						
	Coefficients	*Standard Error*	*t Stat*	*P-value*	*Lower 95%*	*Upper 95%*	*Lower 95.0%*	*Upper 95.0%*
Intercept	0.588655069	0.212711034	2.7673932	0.069720099	-0.088286375	1.265596514	-0.088286375	1.265596514
PMI /days	-8.26864E-05	0.000685911	-0.120549738	0.911668197	-0.002265562	0.002100189	-0.002265562	0.002100189

Amide to phosphate ratio

Humans

sample	PMI /days	Human Inner		PMI /days	Human Outer
HB19	463	0.58		463	0.51
HB25	463	0.62		463	1.58
HB27	463	0.72		463	0.58
HB29	463	0.83		463	0.52
HB37	531	0.49		531	0.50
HB35	714	0.61		714	0.38
HB22	717	0.88		717	0.47
HB23	718	0.57		718	0.54
HB17	725	0.50		725	0.88
HB33	830	0.41		830	0.53
HB13	836	0.57		836	0.40
HB15	949	0.56		949	0.35
HB11	1057	0.70		1057	0.40
HB07	1062	0.63		1062	0.58
HB09	1063	0.63		1063	0.43
HB05	1238	0.93		1238	0.38
HB04	1238	0.61		1238	0.41

Human Inner

Regression Statistics	
Multiple R	0.130482734
R Square	0.017025744
Adjusted R Square	-0.048505873
Standard Error	0.141354079
Observations	17

ANOVA

	df	SS	MS	F	Significance F
Regression	1	0.005191249	0.005191249	0.259809608	0.617667907
Residual	15	0.299714633	0.019980976		
Total	16	0.304905882			

	Coefficients	Standard Error	t Stat	P-value	Lower 95%	Upper 95%	Lower 95.0%	Upper 95.0%
Intercept	0.584517654	0.109726822	5.327026201	8.45955E-05	0.35064047	0.818394838	0.35064047	0.818394838
PMI /days	6.67553E-05	0.000130966	0.509715223	0.617667907	-0.000212392	0.000345903	-0.000212392	0.000345903

Human Outer

Regression Statistics	
Multiple R	0.442003044
R Square	0.195366691
Adjusted R Square	0.141724471
Standard Error	0.270081384
Observations	17

ANOVA

	df	SS	MS	F	Significance F
Regression	1	0.265664224	0.265664224	3.642032138	0.075663341
Residual	15	1.094159306	0.072943954		
Total	16	1.359823529			

	Coefficients	Standard Error	t Stat	P-value	Lower 95%	Upper 95%	Lower 95.0%	Upper 95.0%
Intercept	0.935365824	0.209652046	4.461515361	0.000457076	0.488503066	1.382228583	0.488503066	1.382228583
PMI /days	-0.000477548	0.000250233	-1.908410893	0.075663341	-0.001010907	5.58117E-05	-0.001010907	5.58117E-05

Pigs

sample	PMI /days	Pig Inner		PMI /days	Pig Outer
PB44	78	0.40		78	0.49
PB42	83	0.40		83	0.43
PB50	237	0.48		237	0.45
PB48	268	0.50		268	0.65
PB40	583	0.54		583	0.57

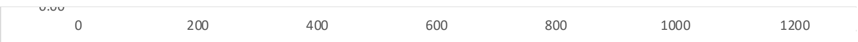

Pig Inner

Regression Statistics	
Multiple R	0.926784439
R Square	0.858929397
Adjusted R Square	0.811905863
Standard Error	0.027014908
Observations	5

ANOVA

	df	SS	MS	F	Significance F
Regression	1	0.013330584	0.013330584	18.26594724	0.023518575
Residual	3	0.002189416	0.000729805		
Total	4	0.01552			

	Coefficients	Standard Error	t Stat	P-value	Lower 95%	Upper 95%	Lower 95.0%	Upper 95.0%
Intercept	0.393812412	0.020387729	19.31614899	0.000303064	0.328929559	0.458695265	0.328929559	0.458695265
PMI /days	0.000280975	6.57426E-05	4.273867948	0.023518575	7.17529E-05	0.000490197	7.17529E-05	0.000490197

Pig Outer

Regression Statistics	
Multiple R	0.534768756
R Square	0.285977622
Adjusted R Square	0.047970163
Standard Error	0.088999372
Observations	5

ANOVA

	df	SS	MS	F	Significance F
Regression	1	0.009517335	0.009517335	1.201548991	0.353122481
Residual	3	0.023762665	0.007920888		
Total	4	0.03328			

	Coefficients	Standard Error	t Stat	P-value	Lower 95%	Upper 95%	Lower 95.0%	Upper 95.0%
Intercept	0.458694713	0.067166436	6.829225146	0.006424059	0.244941137	0.672448289	0.244941137	0.672448289
PMI /days	0.000237411	0.000216586	1.096151901	0.353122481	-0.000451862	0.000926684	-0.000451862	0.000926684

Regression analysis

p-Value

	Human Inner	Human Outer	Pig Inner	Pig Outer
CP	0.617	0.131	0.421	0.912
CI	0.219	0.658	0.264	0.798
N/P	0.618	0.076	0.024	0.353

R^2

	Human Inner	Human Outer	Pig Inner	Pig Outer
CP	0.017	0.146	0.223	0.005
CI	0.099	0.013	0.386	0.025
N/P	0.017	0.195	0.859	0.286
